# H3N2 Influenza Infection Elicits More Cross-Reactive and Less Clonally Expanded Anti-Hemagglutinin Antibodies Than Influenza Vaccination

**DOI:** 10.1371/journal.pone.0025797

**Published:** 2011-10-19

**Authors:** M. Anthony Moody, Ruijun Zhang, Emmanuel B. Walter, Christopher W. Woods, Geoffrey S. Ginsburg, Micah T. McClain, Thomas N. Denny, Xi Chen, Supriya Munshaw, Dawn J. Marshall, John F. Whitesides, Mark S. Drinker, Joshua D. Amos, Thaddeus C. Gurley, Joshua A. Eudailey, Andrew Foulger, Katherine R. DeRosa, Robert Parks, R. Ryan Meyerhoff, Jae-Sung Yu, Daniel M. Kozink, Brice E. Barefoot, Elizabeth A. Ramsburg, Surender Khurana, Hana Golding, Nathan A. Vandergrift, S. Munir Alam, Georgia D. Tomaras, Thomas B. Kepler, Garnett Kelsoe, Hua-Xin Liao, Barton F. Haynes

**Affiliations:** 1 Duke Human Vaccine Institute, Duke University Medical Center, Durham, North Carolina, United States of America; 2 Department of Pediatrics, Duke University Medical Center, Durham, North Carolina, United States of America; 3 Department of Medicine, Duke University Medical Center, Durham, North Carolina, United States of America; 4 Department of Pathology, Duke University Medical Center, Durham, North Carolina, United States of America; 5 Department of Surgery, Duke University Medical Center, Durham, North Carolina, United States of America; 6 Department of Immunology, Duke University Medical Center, Durham, North Carolina, United States of America; 7 Department of Biostatistics & Bioinformatics, Duke University Medical Center, Durham, North Carolina, United States of America; 8 Department of Molecular Genetics & Microbiology, Duke University Medical Center, Durham, North Carolina, United States of America; 9 Institute for Genome Sciences & Policy, Duke University Medical Center, Durham, North Carolina, United States of America; 10 Division of Infectious Diseases, Durham Veteran's Affairs Medical Center, Durham, North Carolina, United States of America; 11 Division of Viral Products, Center for Biologics Evaluation and Research, Food and Drug Administration, Bethesda, Maryland, United States of America; University of Rochester School of Medicine, United States of America

## Abstract

**Background:**

During the recent H1N1 influenza pandemic, excess morbidity and mortality was seen in young but not older adults suggesting that prior infection with influenza strains may have protected older subjects. In contrast, a history of recent seasonal trivalent vaccine in younger adults was not associated with protection.

**Methods and Findings:**

To study hemagglutinin (HA) antibody responses in influenza immunization and infection, we have studied the day 7 plasma cell repertoires of subjects immunized with seasonal trivalent inactivated influenza vaccine (TIV) and compared them to the plasma cell repertoires of subjects experimentally infected (EI) with influenza H3N2 A/Wisconsin/67/2005. The majority of circulating plasma cells after TIV produced influenza-specific antibodies, while most plasma cells after EI produced antibodies that did not react with influenza HA. While anti-HA antibodies from TIV subjects were primarily reactive with single or few HA strains, anti-HA antibodies from EI subjects were isolated that reacted with multiple HA strains. Plasma cell-derived anti-HA antibodies from TIV subjects showed more evidence of clonal expansion compared with antibodies from EI subjects. From an H3N2-infected subject, we isolated a 4-member clonal lineage of broadly cross-reactive antibodies that bound to multiple HA subtypes and neutralized both H1N1 and H3N2 viruses. This broad reactivity was not detected in post-infection plasma suggesting this broadly reactive clonal lineage was not immunodominant in this subject.

**Conclusion:**

The presence of broadly reactive subdominant antibody responses in some EI subjects suggests that improved vaccine designs that make broadly reactive antibody responses immunodominant could protect against novel influenza strains.

## Introduction

Influenza is a persistent threat to public health with seasonal influenza causing >200,000 hospitalizations and >35,000 deaths in the US annually [1], [2]. While the most recent pandemic strain did not appear to be significantly more pathogenic than the seasonal strain of influenza that it replaced [3], prior pandemics, such as the 1918 H1N1 influenza pandemic, have been associated with severe mortality [4].

Immunization of susceptible populations is one of the primary methods for preventing influenza-associated morbidity and mortality [5]. In humans, boosting immunizations with trivalent inactivated influenza vaccine (TIV) are associated with the transient appearance of influenza-specific plasma cells/plasmablasts (hereafter termed plasma cells) in peripheral blood [6]. The majority of these plasma cells produce antibodies that bind HA and are both strain-specific and neutralizing [6]. Protective humoral responses to influenza are mediated by antibodies that prevent infection of target cells, and these antibodies are largely directed against variable regions of the HA globular head leading to subtype- and strain-specific antibody responses [7], [8]. Broadly neutralizing antibodies reactive with multiple influenza subtypes have been isolated from phage-displayed libraries from uninfected subjects [9], those recovering from H5N1 influenza [10], and those vaccinated against seasonal influenza [11], but such antibodies are not immunodominant and generally are not found in plasma [12].

In order to perform a direct comparison between the antibody repertoires following influenza immunization and infection, we isolated plasma cells from human peripheral blood at seven days following TIV or experimental influenza infection (EI) with H3N2 A/Wisconsin/67/2005 by using single cell sorting. PCR-based amplification of V(D)J gene rearrangements of Ig heavy- and light-chains present in single plasma cells was used for analysis and gene recovery for subsequent mAb expression. We found that plasma-cell-derived mAbs from EI were more polyclonal but anti-HA mAbs from EI were more cross-reactive compared to mAbs derived from TIV subjects. The anti-HA response in TIV showed more evidence of clonal expansion and was more strain-specific compared to the response in EI. The largest clonal lineage identified from an EI subject contained anti-HA mAbs that reacted with most HAs tested and neutralized both H1N1 and H3N2 influenza A strains.

## Results

### Similar Frequencies of Circulating Plasma Cells Following TIV and EI

We studied a group of five subjects immunized with TIV and six subjects enrolled in a protocol of EI with influenza H3N2 A/Wisconsin/67/2005 [13] (Table 1). At 21 days after immunization, all TIV subjects showed a >4-fold rise in antibody titer for HA binding for those components in the vaccine (Fig. S1 online) and a rise in influenza neutralization titer vs. H1N1 A/Solomon Islands/03/2006 or H3N2 A/Wisconsin/67/2005 (Table 1). At 28 days after experimental infection, 5/6 EI subjects had a >4-fold rise in antibody titer against the infecting strain H3N2 A/Wisconsin/67/2005 (Fig. S1 online). For one subject, EI03, no convalescent sample was available; testing of the day 7 sample showed a 3.7-fold rise in titer against the infecting strain (Fig. S1 online). Neutralization titers rose for all EI subjects [2-fold to 16-fold rise; Table 1]. Symptom severity did not correlate with infecting dose (Table 1).

**Table 1:** Subject Characteristics.

Subject ID	Age	Gender	Immunogen	Peak Sx Score†	Change in Neutralization Titer from d0 to d21	
					vs. H1 SI¶	vs. H3 Wisc
TIV01	18	Male	TIV 2007*	N/A	1∶20 to >1∶1280	1∶160 to 1∶1280
TIV04	42	Male	TIV 2007	N/A	0 to 1∶240	0 to 1∶320
TIV14	37	Male	TIV 2008*	N/A	1∶80 to 1∶80 (no Δ)	0 to 1∶40
TIV21	26	Male	TIV 2008	N/A	0 to 1∶240	0 to 1∶160
TIV24	20	Female	TIV 2008	N/A	1∶80 to >1∶1280	1∶10 to 1∶640
mean	28.6					

Subject ID	Age	Gender	Infecting Strain	Peak Sx Score†	Change in Neutralization Titer from d0 to d28	
					vs. H1 SI¶	vs. H3 Wisc
EI02	28	Male	H3 Wisc 10^6^ TCID_50_ ‡	0	1∶40 to 1∶40 (no Δ)	1∶10 to 1∶40
EI03	24	Male	H3 Wisc 10^6^ TCID_50_	0	not done	0 to 1∶10§
EI05	25	Female	H3 Wisc 10^5^ TCID_50_	12	1∶10 to 1∶10 (no Δ)	1∶20 to 1∶320
EI07	35	Male	H3 Wisc 10^5^ TCID_50_	12	0 to 0 (no Δ)	0 to 1∶80
EI12	27	Male	H3 Wisc 10^4^ TCID_50_	4	1∶40 to 1∶40 (no Δ)	1∶10 to 1∶80
EI13	29	Female	H3 Wisc 10^3^ TCID_50_	2	not done	1∶40 to 1∶80
mean	28.0					

*TIV 2007 = inactivated trivalent influenza vaccine for 2007–2008 season; TIV 2008 = inactivated trivalent influenza vaccine for 2008–2009 season.

† Modified Jackson score [48]. N/A = not applicable.

¶ H1 SI = H1N1 A/Solomon Islands/03/2006; H3N2 Wisc = H3N2 A/Wisconsin/67/2005.

‡ Number shown is relative inoculum.

§ Sample is from d7; d28 sample not available.

**Figure 1:**
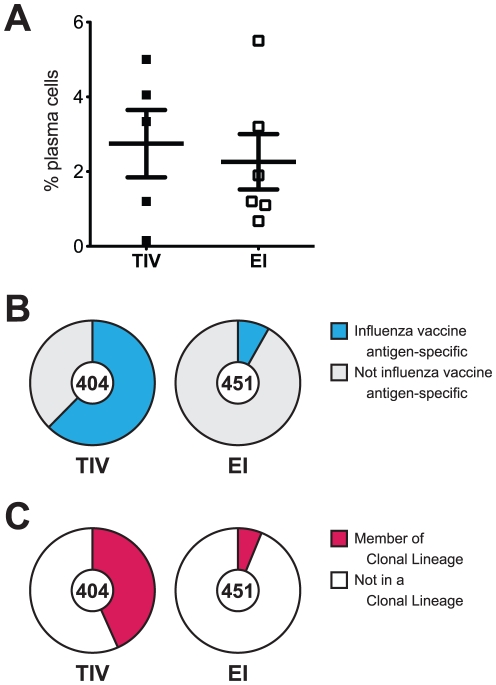
Characterization of peripheral blood plasmacytosis 7 days after TIV or EI. A. Peripheral blood B cells (CD3/14/16/235a^−^ CD19^+^) stained for plasma cell markers (CD3/14/16/235a^−^ CD19^+^ CD20^−/lo^ CD27^hi^ CD38^hi^); points shown are percentage of B cells that were plasma cells. TIV mean 2.75%±0.90%; EI mean 2.26±0.74%; two-tailed *t* test, *p* = 0.68. B. Human rmAbs derived from sorted plasma cells tested for reactivity. From TIV subjects, 252/404 rmAbs (62.4%) reacted with ≥1 influenza antigen (blue wedge), 152/404 (37.6%) did not react with any tested rHA or split-virus antigen (gray wedge). From EI subjects, 37/451 rmAbs (8.2%) reacted with ≥1 influenza antigen (tested vs. TIV, χ^2^ = 279.5, *p*<0.0001), 414/451 (91.8%) did not react with any tested rHA or split-virus antigen. C. Human rmAbs membership in clonal lineages. From TIV subjects, 175/404 (43.3%) rmAbs were members of 46 clonal lineages (red wedge); from EI subjects, 28/451 (6.2%) rmAbs were members of 12 clonal lineages (χ^2^ = 162.1, *p*<0.0001).

As described [14] we analyzed PBMC for the presence of plasma cells (CD3/14/16/235a^−^ CD19^+^ CD20^−/lo^ CD27^hi^ CD38^hi^) seven days after TIV or EI. There was no difference in plasma cell frequencies between five TIV subjects and six EI subjects as a percentage of the total B cell population (CD3/14/16/235a^−^ CD19^+^) in PBMC [TIV mean 2.75%±0.90%; EI mean 2.26±0.74%; two-tailed *t* test, *p* = 0.68] (Fig. 1A; Fig. S2 online).

### Higher Frequencies of Influenza-Specific Plasma Cells After TIV

We isolated single plasma cells from both TIV and EI subjects for the generation of recombinant (r) mAb [14], recovering 404 mAbs from five TIV subjects and 451 mAbs from six EI subjects (Figs. 1B, 1C; Fig. S3 online). The characteristics of all recovered rmAbs are displayed in Tables S3, S4, S5, S6, S7, S8, S9, S10 and Figures S6, S7 online. All 855 rmAbs were tested in ELISA and Luminex®-based assays for reactivity with a panel of nine baculovirus-expressed rHAs and split-virus vaccine preparations (TIV for 2007–2008 or 2008–2009 seasons; see Methods). Split-virus vaccines contain immunogenic HA as well as neuraminidase, nucleoprotein, matrix proteins, and membrane fragments [15]. Almost two-thirds (252/404 rmAbs; 62.4%) of TIV rmAbs reacted with TIV or rHA while fewer than 10% (37/451 rmAbs: 8.2%) of rmAbs from EI subjects did (χ^2^ = 279.5, *p*<0.0001) (Fig. 1B). Thus, while TIV and EI were associated with a similar degree of plasmacytosis, the circulating plasma cells at 7 days in EI were less frequently HA-specific.

### Increased Clonal Relatedness After TIV

We next analyzed the Ig heavy chain (HC) and light chain (LC) gene sequences from all 855 mAbs for clonal relatedness and found that mAbs from TIV subjects were more likely to fit in clonal lineages than mAbs from EI subjects (Fig. 1C; Table S1, Table S2, Fig. S4 online). From TIV subjects, 175/404 (43.3%) mAbs could be arranged into 46 clonal lineages containing 2–19 unique members (Table S1 online). In contrast, 28/451 (6.2%) mAbs from EI subjects could be arranged into one of 12 clonal lineages containing 2–4 unique members (Table S2 online) (Fig. 1C) (χ^2^ = 162.1, *p*<0.0001). Clonal expansion was detected in most subjects [4/5 (80%) of TIV subjects; 4/6 (67%) of EI subjects]; TIV14, EI02, and EI07 lacked evidence for clonal expansion.

**Figure 2:**
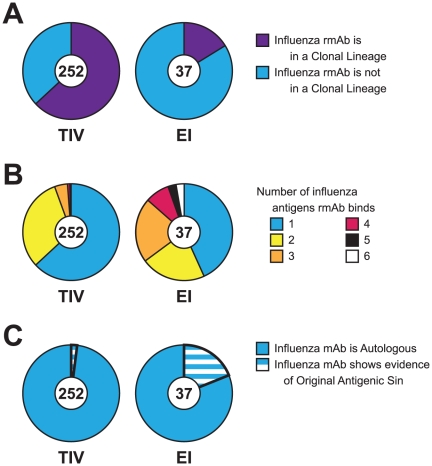
Characterization of influenza-specific mAbs from TIV or EI subjects. A. Clonal lineages. From TIV subjects, 159/252 (63.1%) of influenza-specific rmAbs were members of 44 clonal lineages (purple wedge); from EI subjects, 6/37 (16.2%) were members of 2 clonal lineages (χ^2^ = 28.9, *p*<0.0001). B, Multiple reactivity to influenza antigens. From TIV subjects, 159/252 (63%) of rmAbs were strain-specific (blue wedge); multiply reactive rmAbs were less common [two antigens 79/252 (31.4%) (yellow wedge); three antigens 11/252 (4.4%) (orange wedge); four antigens 2/252 (0.8%) (red wedge); five antigens 1/252 (0.4%) (black wedge)]. From EI subjects, 16/37 (43.2%) were strain-specific (χ^2^ = 7.74, *p* = 0.0054); multiply reactive mAbs were more common [two antigens 8/37 (21.6%); three antigens 8/37 (21.6%); four antigens 3/37 (8.1%); five antigens 1/37 (2.7%); six antigens 1/37 (2.7%) (white wedge)]. C. Original antigenic sin rmAbs. From TIV subjects, 5/252 (2%) of influenza-specific rmAbs did not react with strains contained in the administered vaccine but only with previously circulating influenza antigens (striped wedge). From EI subjects, 7/37 (19%) of influenza-specific rmAbs did not react with the infecting strain but only with previously circulating antigens (χ^2^ = 19.2, *p*<0.0001).

Of the 46 clonal lineages identified from TIV subjects, 44 (96%) had at least one rmAb that reacted with influenza antigens; clonal lineages containing rmAbs reactive with influenza antigens were found in all four TIV subjects with identified clonal lineages (Table S1 online). In contrast, of the 12 clonal lineages recovered from EI subjects, only 2 (17%) contained members reactive with rHAs and split-virus vaccine preparations (Table S2 online). Both influenza-reactive lineages were from one subject (EI13) and all rmAbs from these two lineages reacted with rHA. Ninety-one percent (159/175) of rmAbs in TIV clonal lineages were reactive with influenza antigens (Table S1 online) while only 21% (6/28) of clonal members from EI subjects were reactive with influenza antigens (Table S2 online) (χ^2^ = 76.5, *p*<0.0001). When viewed as a proportion of all rHA-specific mAbs, antibodies from TIV were more likely to be in a clonal lineage (159/252; 63.1%) compared to mAbs from EI (6/37; 16.2%) (Fig. 2A; Fig. S4 online) (χ^2^ = 28.9, *p*<0.0001). Thus, while EI and TIV were associated with a similar degree of plasmacytosis, TIV was associated with more clonal expansion of plasma cells making anti-HA antibodies.

### Cross-reactive rmAbs Recovered from TIV and EI

Using a panel of 9 rHAs representing diverse temporal (1994–2009) and antigenic (influenza A H1, H3, H5 and influenza B) strains, we tested all 855 rmAbs for their ability to bind rHAs in ELISA and Luminex® assays. In addition we tested mAbs for binding to split-virus TIV preparations from the 2007–2008 and 2008–2009 seasons. The majority of influenza-specific mAbs isolated from TIV subjects were strain-specific (159/252; 63%) while rmAbs reactive with single influenza HA strains were less common in EI subjects (16/37; 43%) (χ^2^ = 7.74, *p* = 0.0054) (Fig. 2B). In contrast, rmAbs cross-reactive with 3 or more influenza HA strains were much less common in TIV compared to EI subjects [14/252 (5.6%) vs. 13/37 (35%), respectively; χ^2^ = 33.3, *p*<0.0001] (Fig. 2B, Table S11 online). We found cross-reactive rmAbs associated with clonal expansion in both groups; 6/14 (43%) cross-reactive rmAbs from TIV were members of four clonal lineages while 4/13 (31%) of cross-reactive rmAbs from EI were in a clonal lineage (Tables S1, S2, S11 online).

### TIV rmAb Reactivity Is Similar to Plasma Antibody Specificities

**Figure 3:**
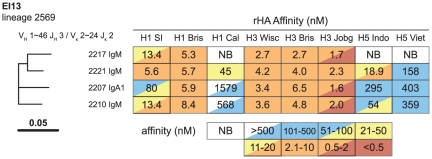
Clonal lineage 2569 from EI13. Three of 4 members (75%) derived from IgM-expressing plasma cells, 1/4 (25%) derived from an IgA1 plasma cell. The highest affinity binding for all members was to H3 Jobg; high affinity binding to other H3 rHAs and H1 Bris was also observed. Three members were tested for HAI and neutralization and displayed similar breadth (Table 3). H1 SI = H1N1 A/Solomon Islands/03/2006; H1 Bris = H1N1 A/Brisbane/59/2007; H1 Cal = H1N1 A/California/04/2009; H3 Wisc = H3N2 A/Wisconsin/67/2005; H3 Bris = H3N2 A/Brisbane/10/2007; H3 Jobg = H3N2 A/Johannesburg/33/1994; H5 Indo = H5N1 A/Indonesia/05/2005; H5 Viet = H5N1 A/Vietnam/1203/2004.

The antibody response in TIV subjects was largely restricted to the subtypes present in the administered vaccine and for most subjects was strain-specific (Text S1, Figs. S1, S8 online). The strain specificity of the response was most striking in the rmAb-binding pattern against rHAs (Text S1, Figs. S1, S8 online). In EI subjects plasma antibody rose more modestly (Fig. S1 online) and recovered rmAbs were less strain specific (Fig. S8 online). The most broadly cross-reactive anti-HA rmAbs were recovered from subject EI13 (Fig. 3; Fig. S8, Table S11 online); comparison of plasma antibody at time zero and 28 days after infection demonstrated a 13-fold increase in rHA H3 A/Wisconsin/67/2005 binding (Fig. S1 online) but only a 2-fold rise in virus neutralization titer to that strain (Table 1). Thus, the broad neutralizing rmAbs isolated from EI13 (Fig. 3) did not contribute significantly to plasma antibody 28 days following EI.

### Higher Frequency of Original Antigenic Sin (OAS) Antibodies in EI

OAS antibodies are defined as rmAbs that react with rHAs to which the subject was exposed before the current vaccine or infection [12], [16], [17], [18], [19]. In this study, OAS rmAbs reacted with rHA strains not contained in the administered vaccine for TIV subjects or with the infecting H3N2 A/Wisconsin/67/2005 strain for EI subjects. From TIV subjects, 5/252 (2%) rmAbs were consistent with OAS (Fig. 2C, Table 2). In contrast, from EI subjects, 7/37 (19%) influenza-specific rmAbs had rHA binding consistent with OAS (Fig. 2C, Table 2); those rmAbs reacted with influenza A H3 rHAs, influenza B rHA, and trivalent vaccine from 2008–2009 (Table 2). Thus, OAS rmAbs from EI subjects comprised a greater proportion of influenza-reactive rmAbs compared to those from TIV subjects (χ^2^ = 19.2, *p*<0.0001) (Fig. 2C).

**Table 2:** Characteristics of OAS rmAbs.

Subject ID	rmAb ID	Lineage*	Isotype	HC Usage	HC CDR3 Length	HC Mutation	LC Usage	LC CDR3 Length	Reactivity†
TIV01	1318	1329	A1	V_H_ 4-30 J_H_ 6	18	2.7%	κ V_κ_ 3-20 J_κ_ 2	10	B Fla, TIV 2008‡
TIV24	2516	N/A	G1	V_H_ 3-15 J_H_ 6	16	10.2%	λ V_λ_ 3-21 J_λ_ 1	11	H3 Wisc, H3 Jobg
TIV24	2523	N/A	G1	V_H_ 3-15 J_H_ 6	16	8.4%	κ V_κ_ 1-33 J_κ_ 5	9	H3 Jobg
TIV24	2549	N/A	G1	V_H_ 3-13 J_H_ 6	21	10.5%	κ V_κ_ 1-39 J_κ_ 4	9	H1 SI
TIV24	2575	N/A	G1	V_H_ 3-15 J_H_ 6	16	4.7%	κ V_κ_ 1-39 J_κ_ 4	9	H3 Jobg
EI03	1938	N/A	G1	V_H_ 4-31 J_H_ 6	19	10.3%	κ V_κ_ 1-39 J_κ_ 5	9	H3 Jobg
EI03	1974	N/A	G1	V_H_ 3-74 J_H_ 4	17	10.3%	κ V_κ_ 3-11 J_κ_ 2	9	H3 Jobg
EI03	1975	N/A	A1	V_H_ 2-70 J_H_ 1	17	5.5%	λ V_λ_ 1-40 J_λ_ 3	11	H3 Jobg
EI03	1997	N/A	G1	V_H_ 1-69 J_H_ 5	14	10.8%	κ V_κ_ 1-13 J_κ_ 4	9	H3 Jobg, H3 Bris
EI13	2318	N/A	A2	V_H_ 1-2 J_H_ 4	16	10.1%	λ V_λ_ 1-51 J_λ_ 2	11	B Fla
EI13	2423	N/A	G1	V_H_ 3-9 J_H_ 4	18	4.8%	κ V_κ_ 1-39 J_κ_ 4	9	H3 Jobg, H3 Bris
EI13	2431	N/A	G1	V_H_ 3-13 J_H_ 4	14	9.4%	λ V_λ_ 1-44 J_λ_ 3	11	H3 Bris

*Lineage ID from Table S1 (available online). N/A = not applicable.

† B Fla = HA B/Florida/04/2006; H3 Wisc = H3 A/Wisconsin/67/2005; H3 Jobg = H3 A/Johannesburg/33/1994; H1 SI = H1 A/Solomon Islands/03/2006; H3 Bris = H3 A/Brisbane/10/2007; TIV 2008 = trivalent influenza vaccine 2008–2009 season.

‡ Reactivity to TIV08 could represent cross-reactivity to the B/Florida/04/2006 component.

Cross-reactive rmAbs (Table S11 online) did not have characteristics of OAS; in fact, no OAS rmAb reacted with more than two rHAs (Table 2). The most common specificity of OAS rmAbs was to rHA H3 A/Johannesburg/33/1994 (TIV 3/5, EI 5/10), a strain isolated more than a decade before the current study was performed. Comparison of the infecting strain with other H3 rHAs tested showed that the Johannesburg strain was least similar (88% amino acid identity vs. 96–98% for other tested strains; Fig. S12 online) suggesting that rHA sequence homology was not responsible for eliciting these antibodies. OAS mAbs were more frequently recovered from the same EI subjects from whom the most broadly cross-reactive mAbs derived, suggesting that these populations were stimulated in parallel during EI. Thus, TIV is characterized by few OAS mAbs, clonal expansion of anti-HA responses, and seroconversion to the vaccine; while EI is characterized by more OAS mAbs, less clonal expansion of anti-HA responses, and weaker seroconversion to the infecting strain.

### Presence of Strain-specific and Cross-reactive Anti-HA rmAbs in a Single Clonal Lineage From an Influenza Vaccinated Subject

**Figure 4:**
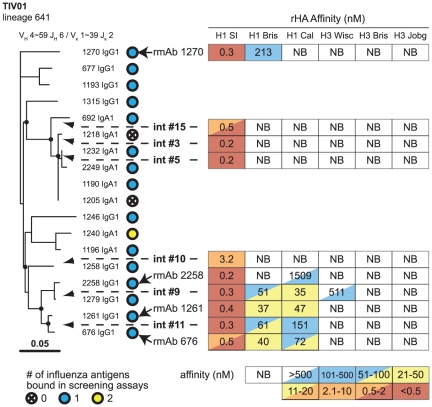
Clonal lineage 641 from TIV01. During screening, 15/18 rmAbs (83%) bound one influenza antigen (blue dots), 1/18 (6%) bound two antigens (yellow dot), 2/18 (11%) bound no antigen tested (crossed dots). Antibody 1270 bound rHA H1 SI with high affinity and H1 Bris with weak affinity. Two branches of the tree derived from IgA1-expressing plasma cells. Inferred intermediates (int) of one of these branches were consistent with affinity maturation (arrows pointing to circles on the tree indicate the position produced int rmAbs); int #15 bound with lower affinity to H1 SI than later int #3 or int #5. Branches of the tree near the bottom showed breadth. Int #10 bound only H1 SI; int #9 had higher affinity for H1 SI, bound H1 Bris and H1 Cal with moderate affinity, and weakly bound H3 Wisc. Int #11 bound H1 Cal more weakly and recovered rmAbs 676 and 1261 bound with a similar pattern. Recovered rmAb 2258 had the highest H1 SI affinity in this part of the lineage but lost cross-reactivity, retaining only weak reactivity to H1 Cal. Embedded tables show affinity measurements in nM for each rmAb; NB = no binding observed. H1 SI = H1N1 A/Solomon Islands/03/2006; H1 Bris = H1N1 A/Brisbane/59/2007; H1 Cal = H1N1 A/California/04/2009; H3 Wisc = H3N2 A/Wisconsin/67/2005; H3 Bris = H3N2 A/Brisbane/10/2007; H3 Jobg = H3N2 A/Johannesburg/33/1994.

Clonal lineages from TIV subjects were commonly recovered and largely influenza strain-specific (Table S1 online). Analysis of lineage 641 recovered from TIV01 showed remarkable divergence in rHA reactivity within the clonal lineage (Fig. 4). This lineage is composed of IgG1 and IgA1 members (10/18 and 8/18, respectively), and rmAbs in the upper portion of the lineage reacted primarily with rHA H1 A/Solomon Islands/03/2006 (15/18, 83%). For example, rmAb 1270 displayed high affinity for H1 A/Solomon Islands/03/2006 and much lower affinity for H1 A/Brisbane/59/2007 (Fig. 4). These data were consistent with the neutralization pattern of rmAb 1270 that inhibited H1N1 A/Solomon Islands/03/2006 at 0.02 µg/mL but did not inhibit H3N2 A/Wisconsin/67/2005.

One of the IgA1 branches of lineage 641 was composed of six members (rmAbs 692, 1190, 1205, 1218, 1232, and 2249), two of which (1205, 1218) did not bind to any rHA tested (Fig. 4). To study affinity maturation in this lineage branch, we produced inferred intermediate antibodies #3 and #5 (Fig. 4) and these bound with high affinity to H1 A/Solomon Islands/03/2006 but did not bind to other rHAs. The more distantly related branch point intermediate #15 also bound only to H1 A/Solomon Islands/03/2006 but with lower affinity (Fig. 4). These data are consistent with affinity maturation occurring within this branch of clone 641.

Remarkably, IgG1 clonal lineage members in the bottom portion of the 641 tree displayed both an increased affinity for rHA H1 A/Solomon Islands/03/2006 and to other rHAs (Fig. 4). In particular, intermediate #9 bound with high affinity to H1 A/Solomon Islands/03/2006, with moderate affinity to both H1 A/Brisbane/59/2007 and pandemic H1 A/California/04/2009, and with weak affinity to H3 A/Wisconsin/67/2005 (Fig. 4). However, this rHA binding breadth was lost in some more distant members of the lineage (*e.g.*, rmAb 2258; Fig. 4). Intermediate #11 did not bind rHA H3 A/Wisconsin/67/2005 and showed weak affinity for novel H1 A/California/04/2009; the two rmAbs derived from this intermediate (rmAbs 1261 and 676) retained similar breadth and affinity of rHA binding as intermediate #11 (Fig. 4). Subject TIV01 from whom this lineage was derived showed a greater rise in plasma H1N1 A/Solomon Islands/03/2006 neutralization (Table 1) and binding titer (Fig. S1 online) compared to the rise against H3N2 A/Wisconsin/67/2005; and the rmAbs in lineage 641 recapitulate this finding.

Overall, nine lineages from TIV subjects showed evidence of class switching with IgG1 to IgA1 class switching being most common (7/9 lineages; Table S1 online). Additional clonal lineages from subjects TIV01 and TIV21 are presented in the supporting materials online (Text S1, Figs. S10 and S11 online).

### Broadly Reactive Clonal Lineage 2569 from H3N2 Infected Subject EI13

As noted, clonal lineages from EI subjects were detected less frequently than from TIV subjects, and only two clonal lineages had rmAbs reactive with influenza antigens (Table S2 online). The largest clonal lineage from subject EI13 consisted of four highly cross-reactive mAbs (Fig. 3, Table 3; Table S11 online). This lineage had one IgA1 and three IgM members; each member of this lineage had a VDJ mutation frequency higher than the average for influenza-specific mAbs from EI (range 8.7–14.4%; EI overall mean 8.6%; Fig. S5 online). Members of this lineage bound rHAs of H1N1 A/Solomon Islands/03/2006 and H3N2 A/Wisconsin/67/2005 in surface plasmon resonance (Fig. S9B, S9C online) and blocked hemagglutination of these two strains (Table 3). These rmAbs also neutralized five temporally distinct H3N2 viruses as well as H1N1 A/Solomon Islands/03/2006 (Table 3). High affinity binding to H3 rHA proteins, lower affinity for H5 rHAs and even lower affinity for rHA of the pandemic H1N1 A/California/04/2009 strain paralleled the observed pattern of neutralizing activity (Table 3, Fig. 3). The highest affinity binding in clonal lineage 2569 was observed to a temporally remote strain, H3 A/Johannesburg/33/1994 (Fig. 3) that has lower sequence homology to the infecting strain than other tested rHAs (Fig. S12 online). As noted above, broadly HA-reactive antibodies did not boost (Fig. S1 online) nor did neutralizing antibodies persist as a high-titered response in this subject (Table 1). These data suggest that in this subject, H3N2 A/Wisconsin/67/2005 stimulated a subdominant clone of B cells capable of making broadly neutralizing antibodies but that those antibodies did not significantly contribute to the plasma antibody pool at day 28 after infection (Table 1; Fig. S1 online).

**Table 3:** Anti-influenza activity of rmAbs from EI13 Clonal Lineage 2569.

rmAb ID	2210	2217	2221
HAI (µg/mL)			
H1 SI*	0.02	0.04	0.02
H3 Wisc	0.02	0.02	0.02
Neutralization (µg/mL)†			
H1 SI	1.4	4.9	6.2
H1 Bris	Neg§	Neg	Neg
H1 Cal	Neg	Neg	Neg
H3 Wisc	0.18	0.61	0.39
H3 Urgy	0.09	0.07	0.1
H3 Vict	0.09	0.19	0.19
H3 NY	0.19	0.15	–
H3 Cal	0.09	0.08	–

*H1 SI = H1N1 A/Solomon Islands/03/2006; H1 Bris = H1N1 A/Brisbane/59/2007; H1 Cal = H1N1 A/California/04/2009; H3 Wisc = H3N2 A/Wisconsin/67/2005; H3 Urgy = H3N2 A/Uruguay/716/2007; H3 Vict = H3N2 A/Victoria/210/2009; H3 NY = H3N2 A/New York/55/2004; H3 Cal = H3N2 A/California/7/2004.

† Neutralization testing of H5N1 A/Indonesia/05/2005 and H5N1 A/Vietnam/1203/2004 was negative.

§ – = Not assayed/data not available. Neg = neutralization not detected above assay threshold.

## Discussion

In this study, we compared the plasma cell repertoires present at 7 days following TIV and EI. Previous studies have shown that influenza-specific plasma cells appear transiently in the blood with a peak at 7 days after immunization following which they rapidly decline [6]. Studies of infection, however, have been limited to natural infection where timing must be estimated based on exposure and symptoms [20], or where the infection occurred months to years before antibodies were isolated [10], [21], [22]. This study of vaccination and experimental infection allowed us to compare the two stimuli at exactly the same point after challenge. We found a similar degree of plasmacytosis in the two conditions but found that the frequency of HA-specific plasma cells and the degree of clonality of those cells was lower in the EI group. These findings differ from studies of natural infection where both a higher frequency and clonality of influenza-specific antibodies were found [20]. Trafficking of B cells after infection may have a different kinetic pattern than that following vaccination. This EI study stopped collecting cell samples at 7 days post-infection and so future studies of later time points will be required to determine if the polyclonality of the plasma cell response we observed is specific for the period early after infection, specific for H3N2 A/Wisconsin/67/2005 infection, or if infection with novel pandemic strains consistently stimulates B cell clonal expansion.

For each subject there was an increase in rHA binding and neutralization titers 3–4 weeks after antigen stimulation. The magnitude of rise was greater in the TIV cohort although all EI subjects showed a rise in titer against the infecting strain that was similar to that observed in outbreak situations [23]. The presence of asymptomatic but productive infection is common and has been observed for seasonal influenza [24], pandemic H1N1 [23], and human infection with avian H5N1 [25]. Thus, we found it interesting that similar frequencies of rHA-specific antibodies were recovered from subjects with the highest and lowest symptom scores (Fig. S3 online), and that the most broadly cross-reactive rmAbs were recovered from a subject with mild symptoms and a modest rise in binding and neutralization titer. Whether this response contributed to the mild symptoms experienced by this subject is unknown and future studies will have to address the potential therapeutic role for this group of rmAbs.

The plasma-cell-derived mAbs from TIV subjects were more frequently rHA-specific and showed evidence of clonal expansion while mAbs derived from EI subjects were less frequently rHA-specific and had less evidence of clonal expansion. In all subjects, anti-HA responses were primarily strain-specific, but mAbs derived from EI subjects were more frequently cross-reactive for multiple influenza strains or consistent with OAS compared with mAbs derived from TIV subjects. In the EI cohort, responses to influenza proteins induced by infection that were not detected in our assays may have been present. Although split-virus vaccines like that we used for screening contains antigens other than HA [15], the presence of antibodies reactive with antigens not tested by our assays cannot be excluded. Isolation of rmAbs from both EI and TIV subjects reactive only with split-virus vaccine preparations (Fig. S8 online) suggests that some recovered rmAbs were reactive with antigens other than HA.

Vaccination is the primary means to prevent seasonal [5] and pandemic [26] influenza infection, however, antibody responses to TIV are generally not broadly-neutralizing but rather strain-specific and directed at highly variable domains on HA [7], [8]. Such responses are not absolute—one rmAb isolated from TIV01 was recently shown to have cross-reactivity related to its ability to recognize the sialic acid receptor-binding pocket of HA [27]. Vaccines that bypass regions of diversity by targeting other influenza proteins [*e.g.*, the virus-associated proton-channel M2 [28]] have not been successful in clinical trials [29]. Targeting conserved regions on HA could provide a route to a “universal” influenza vaccine [8], [30], [31], but seasonal vaccines have not consistently induced these specificities of antibodies as shown in our study. Influenza vaccination strategies using novel adjuvants may induce a greater antibody breadth against HA [32] and it will be of interest to compare mAbs derived from newer vaccines with those currently in use.

By producing inferred intermediate antibodies of one clonal lineage, we found evidence of induction and modulation of antibody breadth stimulated by TIV (clonal lineage 641; Fig. 4). This evidence of affinity maturation in humans is similar to findings in mice [33], [34] that were used as evidence of V(D)J mutation as the source of affinity maturation [35]. Clonal lineage 641 was remarkable in that some inferred intermediate rmAbs showed a greater breadth of rHA binding than the recovered rmAbs, suggesting that continued affinity maturation might have eliminated rHA cross-reactivity. Our novel observation of a branch displaying cross-reactivity in an otherwise strain-specific clonal lineage implies that making such cross-reactive branches immunodominant through novel vaccine strategies could lead to improved cross-strain protection.

In contrast to the findings in TIV subjects, we found that EI subjects showed less evidence of clonal expansion seven days after infection despite having plasma cell antibodies with a higher frequency of VDJ mutations. Our observation of polyclonal activation after EI is similar to that seen in animals after γ-herpesvirus infection [36]. This suggests that in our EI subjects either B cell clonal expansion was not required for circulation of B cells at seven days after infection expressing affinity matured cross-reactive antibodies, and/or that the circulation dynamics of HA-specific plasma cells seven days after infection was different than that observed after vaccination.

The human mAb response to influenza infection has been studied in survivors of H5N1 avian influenza [10], [22] and in isolation of mAbs from survivors of the 1918 H1N1 pandemic [21]; in these studies some mAbs were cross-reactive with related strains [21] and some mAbs also displayed cross-protection for novel influenza strains [22], [37]. While broadly cross-reactive neutralizing antibodies against influenza can be induced with TIV, these responses are not sufficiently immunodominant to result in high-titer neutralizing antibodies that provide protection against infection with highly divergent strains. Our data demonstrate this phenomenon directly, in that cross-reactive rmAbs and inferred intermediates were found in the plasma cell repertoires of both TIV and EI subjects while such cross-reactive antibodies were less prominent or not detected in day 21 or day 28 plasma samples.

Work in mice suggested that some anti-influenza responses can be associated with restriction of Ig gene usage [38], and recent work has suggested that human antibodies with broad cross-reactivity may preferentially derive from restricted Ig gene pools [10], [11], [36]. Phage-displayed antibody libraries screened for binding to H5 HA were enriched for V_H_1-69 usage [11] and broadly reactive anti-HA stem mAbs using V_H_1-69 and V_H_3-21 have been produced from IgM^+^ memory B cells [39]. Corti et al. recently reported a series of TIV-induced cross-reactive antibodies using primarily V_H_1-69 demonstrating that cells expressing those antibodies can be isolated following seasonal vaccination [12]; a strict requirement for V_H_1-69 was not shown as there were additional antibodies using V_H_3-23, V_H_3-30, V_H_3-53 and V_H_4-39. It was interesting that none of our clonal lineages used V_H_1-69 and only three cross-reactive antibodies in this study used that V_H_ gene. This could be due to a lack of V_H_1-69 gene usage in our subject population as the frequency of V_H_1-69 expressing B cells is related to copy number variation [40], and whether this is the case for our current subject groups is not known. In our study, other Ig genes were found to make cross-reactive antibodies including V_H_3-23, V_H_3-74, and V_H_1-46. One group of these antibodies, clonal lineage 2569, was remarkable both in the degree of mutation and that its members were IgM and IgA1. These findings are consistent with the recall of an IgM^+^ memory B cell lineage expressing a broadly cross-reactive antibody response that appeared transiently following infection but that did not predominate in plasma four weeks after infection. A similar phenomenon has been observed following TIV with isolation of broadly reactive subdominant antibodies that did not predominate in convalescent plasma [12]. In our EI subject, stimulation of a subdominant response may have contributed to the mild symptoms experienced by this subject and could reflect one mechanism by which antibody responses contribute to control of a virus infection without resulting in long-lasting antibody titers. These findings are also consistent with Pape et al. who reported that class-switched memory B cells have different circulation kinetics compared to IgM memory B cells, with the latter persisting for longer periods but also being subject to suppression in the presence of cross-reactive plasma antibodies [41].

Finally, OAS antibodies were more frequent among mAbs derived from EI subjects compared with TIV subjects. Prior studies have shown that OAS occurs following both influenza vaccination [18] and infection [16], [19], although work by Wrammert et al. suggested that OAS in humans following influenza vaccination was uncommon; our study of TIV subjects is consistent with Wrammert et al. [6]. That the majority of OAS mAbs were reactive with HA from the H3 A/Johannesburg/33/1994 strain is consistent with both prior exposure to that strain and recall by immunization with H3N2-containing vaccine or by infection with H3N2 influenza virus.

Regardless, the presence of both OAS and cross-reactivity in mAbs from EI subjects suggests that both kinds of antibodies were stimulated in parallel during infection. Whether these two processes can be decoupled to only stimulate broadly cross-reactive antibodies by a vaccine remains unknown. Corti et al. showed that the H5 anti-HA antibody response following TIV was detectable but generally weak, both for serum antibodies and memory B cell frequency [12]. Recent reports have described vaccine designs that may stimulate more broadly reactive anti-HA antibodies. Khurana et al. showed increased anti-HA antibody breadth using an adjuvanted vaccine [32]. Wei et al. [31] and Wang et al. [42] have demonstrated induction of broad of plasma antibody responses by sequential immunization with heterologous HAs, perhaps replicating the cross-protective response that can occur after multiple seasonal influenza infections in ferrets [43]. The fact that both seasonal influenza vaccination and infection involve exposure to heterologous HAs on an annual basis, and that these exposures do not lead to high levels of influenza resistance in the general population, suggests that sequential exposure to heterologous HAs by itself is insufficient. Targeting of the conserved HA stalk to induce antibodies in mice reactive across influenza subtypes has recently been demonstrated through the use of synthetic peptides [44] and rHA subunits [45]. It remains to be seen whether such vaccine strategies will work in humans.

In summary, we have shown that while the anti-HA response in vaccination and H3N2 A/Wisconsin/67/2005 infection differs in the degree of clonal expansion present at seven days, broadly reactive antibodies are induced in both settings. One strategy for vaccine design to expand B cell clonal lineages of broadly cross-reactive antibodies may be to create novel HA molecules with enhanced binding to germline B cell receptors of HA-responsive naïve B cells [46], [47]. Thus, the study of clonal lineages of antibodies with HA binding and breadth of influenza neutralization in both vaccination and infection could provide guidance for design of influenza vaccines capable of inducing immunodominant broadly cross-reactive antibody responses.

## Methods

### Ethics Statement

All subject recruitment was performed using written informed consent. For studies performed at Duke University, the Duke University Health System Institutional Review Board for Clinical Investigations approved the protocols. For studies performed at Retroscreen Virology, LTD (Brentwood, UK), the protocol was approved by the East London and City Research Ethics Committee 1 (London, UK), an independent institutional review board (WIRB: Western Institutional Review Board; Olympia, WA), the Duke University Health System Institutional Review Board for Clinical Investigations (Durham, NC), and the SSC-SD IRB (US Department of Defense; Washington, DC). Subjects recruited at Retroscreen Viorology, LTD also consented to unspecified future use of their specimens; these specimens were kept at Duke University in the Clinico-Molecular Predictors of Presymptomatic Infectious Disease – Biorepository. The research performed was approved by the Duke University Health System Institutional Review Board for Clinical Investigations either as a part of the original study or as an exempt study via the biorepository.

### Subjects

Subjects in the TIV group were recruited at Duke University and were given trivalent inactivated seasonal influenza vaccine (Sanofi Pasteur, Swiftwater PA): 2007–2008 Fluzone® vaccine containing A/Solomon Islands/3/2006 (H1N1), A/Wisconsin/67/2005 (H3N2), and B/Malaysia/2506/2004 was given to TIV01 and TIV04; 2008–2009 Fluzone® vaccine containing A/Brisbane/59/2007 (H1N1), A/Uruguay/716/2007 (H3N2) (classified as a A/Brisbane/10/2007-like strain), and B/Florida/04/2006 was given to TIV14, TIV21, and TIV24. Blood was drawn from immunized subjects on day 0 before vaccination and on days 7 and 21 after challenge.

The EI protocol was performed at Retroscreen Virology, LTD (Brentwood, UK) as previously described [13]. Subjects were prescreened and provided informed consent before being given an intranasal challenge. The influenza challenge stock was A/Wisconsin/67/2005 (H3N2) manufactured from a human isolate passaged three times in Primary Chick cells, then twice in eggs, before two final passages in GMP Vero cells. The latter stage of passaging in GMP Vero cells was accomplished under GMP conditions at Baxter BioScience (Vienna, Austria). The final product underwent quality testing for identity, appearance, sterility, infectivity and adventitious contaminants, and was assessed according to pre-determined specifications. On the day of inoculation, volunteers received 10^6.41^ (subjects EI02, EI03), 10^5.25^ (subjects EI05, EI07), 10^4.41^ (subject EI12), and 10^3.08^ (subject EI13) TCID_50_ of the challenge stock. In this protocol, blood was drawn before challenge, then daily on days 0–7, and on day 28 after challenge. Cells were only collected on samples through day 7. Symptoms were recorded twice daily using a modified Jackson scoring system [48]. Infection was confirmed in all subjects by the presence of viral shedding in nasal washings, seroconversion in day 28 serum samples, or both.

### Single-Cell Sorting

PBMC were isolated from blood on the day of draw and cryopreserved using standard techniques until thawed for sorting. Cryopreservation of PBMC was necessary as one protocol was carried out in the UK; in order to harmonize the two protocols all samples were studied after cryopreservation. Single-cell sorting was performed as previously described [14] using a panel of antibodies reactive with the following cell surface molecules: CD138 (FITC), surface IgD (PE), CD3 (PE-Cy5), CD16 (PE-Cy5), CD235a (PE-Cy5), CD20 (PE-Cy7), CD19 (APC-Cy7), CD27 (PacificBlue®) (BD Biosciences, San Jose, CA); CD38 (APC-Cy5.5) (eBioscience, San Diego, CA); CD14 (PE-Cy5), CD38 (APC-Cy5.5) (Invitrogen, Carlsbad, CA); CD38 (APC-AlexaFluor® 647) (Beckman Coulter, Brea, CA). Plasma cells/plasmablasts were sorted using a BD FACSAria™ or a BD FACSAria™ II (BD Biosciences, San Jose, CA) by gating on CD3^−^ CD14^−^ CD16^−^ CD235a^−^ CD19^+^ CD20^−/lo^ CD27^hi^ CD38^hi^ cells. Flow cytometry data was analyzed using FlowJo (Treestar, Ashland, OR). The fraction of plasma cells isolated from peripheral blood is lower than the level reported by Wrammert et al. [mean 6.4%] [6]; this is likely due to the use of cryopreserved PBMC in the present study.

Single cells were directly sorted into 96-well plates containing 20 µL per well of RT reaction buffer [5 µL of 5× first strand cDNA buffer, 0.5 µL RNaseOUT™ (Invitrogen, Carlsbad, CA), 1.25 µL dithiothreitol, 0.0625 µL IGEPAL® CA-630 (Sigma, St. Louis, MO), 13.25 µL of dH_2_O (Invitrogen, Carlsbad, CA)]; plates were stored at −80°C until use and again stored at −80°C until PCR was performed.

### PCR Isolation of Ig V_H_, V_κ_ and V_λ_ Genes

Single cell PCR was achieved as described [6], [14], [49]. Briefly, reverse transcription (RT) was performed using 50 units per reaction Superscript III reverse transcriptase (Invitrogen, Carlsbad, CA) and 0.5 µM human constant region primers (IgG, IgA1, IgA2, IgM, IgD, Igκ, Igλ) at 37°C for 1 h. Separate reactions were used to amplify individual families of V_H_, V_κ_, and V_λ_ genes from the cDNA template; this was performed using two rounds of PCR [first round: 5 µL of RT reaction product, 5 units HotStar Taq Plus (QIAGEN, Valencia, CA), 0.2 mM dNTPs, 0.5 µM nested constant region primers (IgH consisting of IgM, IgD, IgG, IgA1, IgA2; Igκ; or Igλ) and matched variable region primers; second round: 2.5 µL of first round reaction product, 5 units HotStar Taq Plus (QIAGEN, Valencia, CA), 0.2 mM dNTPs, 0.5 µM nested constant region and nested variable region primers]. First round PCR was cycled as follows: 95°C×5 min, 35 cycles of [95°C×30 s, 55°C (V_H_ and V_κ_) or 50°C (V_λ_)×60 s, 72°C×90 s] and one cycle 72°C×7 min. Second round PCR was similar except the extension step was performed at 58°C (V_H_), 60°C (V_κ_), or 64°C (V_λ_). Products were analyzed using agarose gels (1.2%) and purified using PCR purification kits (QIAGEN, Valencia, CA).

Products were sequenced in forward and reverse directions using a BigDye® sequencing kit on an ABI 3700 (Applied Biosystems, Foster City, CA). Sequence base calling was performed using Phred [50], [51]; forward and reverse strands were assembled using an assembly algorithm based on the quality scores at each position [52]. The estimated PCR artifact rate was 0.28 or approximately one PCR artifact per five genes amplified. Ig isotype was determined by local alignment with genes of known isotype [53]; V, D, and J region genes, CDR3 loop lengths, and mutation frequencies were identified using SoDA [54]. All data was annotated so that matching subject data and sort information was linked to the cDNA sequence and analysis results.

For this analysis, when two LC were paired with a single HC (55 from TIV, 60 from EI) the LC with the higher estimated frequency of mutation as calculated by SoDA was selected for inclusion in the dataset. This resulted in unambiguous selection in 114 cases. In one case both LC had an estimated rate of mutation of 0%; in this case the LC was selected at random. Both possible members of this pair were tested for binding and neither pairing was found to be reactive with any antigen tested.

### Clonal lineage determination

Antibody gene sequences from individual subjects were grouped and analyzed using the following criteria: 1) matching of variable and joining region gene segments; 2) matching of CDR3 loop lengths; and 3) ≥70% homology in CDR3 nucleotide sequence. Clonal lineages were only identified if both heavy and light chains for a given group satisfied all three criteria. For heavy chain alignments, D region usage was not considered; *i.e.*, heavy chain genes could be considered part of a clonal lineage if they satisfied the above three criteria but had different predicted D region gene usage. Maximum likelihood trees for clonal lineages were generated using V(D)J regions (excluding constant region sequences); trees were constructed (dnaml), reorganized (retree), and plotted (drawgram) with the PHYLIP 3.69 package [55]. Inferred intermediate V_H_ and V_L_ sequences generated by dnaml were synthesized (GeneScript, Piscataway, NJ) and expressed as IgG1 rmAbs as described below.

### Expression of V_H_ and V_L_ as full-length IgG1 recombinant mAbs

Isolated Ig V_H_ and V_L_ gene pairs were assembled by PCR into linear full-length Ig heavy- and light-chain gene expression cassettes using methods as described [14]. Human embryonic kidney cell line 293T (ATCC, Manassas, VA) was grown to near confluence in 6-well tissue culture plates (Becton Dickinson, Franklin Lakes, NJ) and transfected with 2 µg per well of both IgH and IgL purified PCR-produced cassettes using PolyFect or Effectene (QIAGEN, Valencia, CA). For PolyFect transfected cells, six to eight hours after transfection cells were fed with fresh culture medium supplemented with 2% fetal bovine serum and were incubated at 37°C in a 5% CO_2_ incubator. For Effectene transfected cells, this wash step was omitted. Culture supernatants were harvested three days after transfection and concentrated four-fold using centrifugal concentrators; expressed IgG was quantitated by ELISA [56]; tested mAbs were expressed at 10 µg/mL up to 20 mg/mL. For larger scale production of recombinant mAbs, some linear IgH and IgL gene constructs were cloned into pcDNA 3.3 using standard molecular protocols.

### Antibody binding by ELISA, Luminex® and indirect immunofluorescence

Plasma samples from both subject cohorts were evaluated by ELISA [57] against a panel of baculovirus-expressed purified hemagglutinins (H1 A/Solomon Islands/03/2006, H1 A/Brisbane/59/2007, H1 A/California/04/2009, H3 A/Wisconsin/67/2005, H3 A/Brisbane/10/2007, H3 A/Johannesburg/33/1994, H5 A/Vietnam/1203/2004, H5 A/Indonesia/05/2005, B/Florida/04/2006) (Protein Sciences, Meridien, CT) and split virus vaccine preparations [2007–2008 Fluzone® vaccine containing A/Solomon Islands/3/2006 (H1N1), A/Wisconsin/67/2005 (H3N2), and B/Malaysia/2506/2004; 2008–2009 Fluzone® vaccine containing A/Brisbane/59/2007 (H1N1), A/Uruguay/716/2007 (H3N2) (classified as a A/Brisbane/10/2007-like strain), and B/Florida/04/2006] (Sanofi Pasteur, Swiftwater PA). Split-virus vaccine products have been previously shown to contain HA, neuraminidase, nucleoprotein, matrix protein, and membrane fragments [15]. Samples were diluted serially for the analysis and data were analyzed using 5-parameter curve fitting; endpoint titers were calculated as 3-fold above assay background. The assay cutoff was a 1∶25 dilution. Expressed mAbs were tested in the same ELISA system. ELISA testing for antigens was considered positive if the optical density reading was above 0.13 units, except in the case of split virus influenza vaccine ELISA which was considered positive if the optical density reading was above 0.20 units. Affinity of rmAbs was determined using 4-parameter curve fit of ELISA titer data.

Reactivity to influenza antigens was also studied using a standardized custom Luminex® assay [58]. Luminex® assays were considered positive if the blank-bead-subtracted value was greater than 20 units and greater than 10 divided by IgG concentration in µg/mL.

Transiently expressed mAbs with low IgG concentration were re-transfected and re-assayed. In cases where multiple tests of the same antibody were available, the final dataset contained data from the assay with the highest concentration of mAb. In order to account for antigen overlap between influenza vaccines and purified HAs, antigen reactivities were condensed if reactivity was found to certain antigen pairs (*e.g.*, mAb reactivity to both influenza vaccine 2007–2008 and H3 A/Wisconsin/67/2005 was counted singly). Antigen pairs treated this way were as follows: 2007–2008 vaccine & H1 A/Solomon Islands/03/2006; 2007–2008 vaccine & H3 A/Wisconsin/67/2005; 2008–2009 vaccine & H1 A/Brisbane/59/2007; 2008–2009 vaccine & H3 A/Brisbane/10/2007; 2008–2009 vaccine & B/Florida/04/2006.

### Surface Plasmon resonance (SPR) analysis of antibody reactivity

SPR binding assays were performed on a BIAcore 3000 (BIAcore Inc, Piscataway, NJ) maintained at 20°C. HA recombinant protein was immobilized on a CM5 sensor chip by standard amine coupling as described [57], [59]. Additional tests were performed by capturing human mAbs on anti-human Fc antibody-coupled surfaces; each human mAb was captured to about 200–500 RU. Specific binding responses of mAb binding were obtained following subtraction of non-specific binding on control surfaces. Rate constants were measured using the bivalent analyte model (to account for the avidity of bivalent Ig molecules) and global curve fitting to binding curves obtained from mAb titrations. Antibodies were injected at 30 µL/min for 2–6 min; glycine-HCl pH 2.0 was used as the regeneration buffer.

### Hemagglutination Inhibition (HAI) Assay and Influenza Microneutralization Assay

The hemagglutination inhibition (HAI) assay and microneutralization assays were based on standard published protocols [60]. Influenza stocks were grown in embryonated eggs and were titered for hemagglutination units on turkey red blood cells. To perform HAI assays, serial dilutions in PBS of plasma or transfected cell supernatants were placed into 96-well plates and were mixed with an equal volume of washed turkey red blood cells (0.5%) and incubated at room temperature for 30 min before hemagglutination was read directly from the wells.

For microneutralization, MDCK cells were cultured as monolayers in 96-well culture plates at 37°C in 5% CO_2_, followed by the addition of mixtures of serial dilutions of plasma or purified mAbs and 100 TCID_50_ of the appropriate infectious influenza virus stock. Plates were incubated at 37°C in 5% CO_2_ for 18 h prior to assay. Final assay of the infectious cultures was performed by using fluorescence (for virus constructs containing fluorescent reporter genes); by ELISA using mouse mAb anti-Influenza A nucleoprotein (BEI resources, Manassas, VA) at 1∶4000 dilution, followed by HRP-conjugated Goat anti-mouse IgG (KPL, Gaithersberg, MD) at 1∶2,500 dilution (according to CDC SOP); or by testing of the resulting virus cultures in HAI as described above.

### Statistical analysis

Statistical tests were performed in SAS v9.2 (SAS Institute, Cary, NC). Comparisons for multiple groups (*i.e.*, CDR3 length, mutation rate) were performed using multiple degree of freedom F-tests using PROC GLM in SAS v9.2 with subsequent pairwise comparisons. For data consisting of two groups only, *t*-tests using the Satterthwaite correction (for continuous variables), the Kolmogorov-Smirnov test (for comparison of probability distributions), and Pearson's chi squared tests (for 2×2 category tables) were performed using the appropriate SAS PROC in SAS v9.2; the statistical test used is noted when *p*-values are presented. Graphs of the data were created using GraphPad Prism (GraphPad Software, La Jolla, CA) with layout in Illustrator CS4 (Adobe, San Jose, CA).

## Supporting Information

Text S1 **Additional description of results presented in Supplemental Information.**
(PDF)

Figure S1 **Influenza antigen binding titers of plasma from TIV and EI subjects.** Plasma samples from day 0 and from day 21 (TIV) or day 28 (EI) were tested by ELISA for binding to split virus vaccine preparations and to purified recombinant hemagglutinins. For subject EI03, no day 28 plasma sample was available; a day 7 plasma sample was substituted for this analysis. ELISA was performed using serial dilutions and optical density readings were fitted to a 5-parameter curve; endpoint titers were determined as three fold over the background of the assay for each run. Data are plotted as reciprocal titer values and are pre-immunization/pre-infection titer (x-axis) vs. post-immunization/post-infection titer (y-axis). The threshold of the assay was 1∶25 dilution; endpoint titers that fell below that cutoff were adjusted to that value. The diagonal line for each plot represents a four-fold rise in titer; distance above the diagonal line is proportional to boosting. Each antigen is represented by a number or letter; these are color coded (blue for antigens contained in the 2007–2008 vaccine, red for antigens in the 2008–2009 vaccine, black for antigens unrelated to either vaccine). The code for the graphs is as follows: 7 = 2007–2008 influenza vaccine (blue); S = H1 A/Solomon Islands/03/2006 (blue); W = H3 A/Wisconsin/67/2005 (blue); 8 = 2008–2009 influenza vaccine (red); Z = H1 A/Brisbane/59/2007 (red); N = H3 A/Brisbane/10/2007 (red); B = B/Florida/04/2006 (red); J = H3 A/Johannesburg/33/1994 (black); I = H5 A/Indonesia/05/2005 (black); V = H5 A/Vietnam/1203/2004 (black); H = H1 A/California/04/2009 (black). For individual subjects, reactivity to specific strains was found to dominate. TIV01: rise against S = H1 A/Solomon Islands/03/2006. TIV04: rise against S = H1 A/Solomon Islands/03/2006. TIV14: No clear dominant response. TIV21: rise against 8 = 2008–2009 influenza vaccine and B = B/Florida/04/2006. TIV24: rise against 8 = 2008–2009 influenza vaccine and Z = H1 A/Brisbane/59/2007. Cross-reactive response to S = H1 A/Solomon Islands/03/2006. EI02: slight rise against W = H3 A/Wisconsin/67/2005 and N = H3 A/Brisbane/10/2007 (points overlap). EI03: data derived from day 0 and day 7 samples, 3.73-fold rise against W = H3 A/Wisconsin/67/2005. EI05: rise against W = H3 A/Wisconsin/67/2005 and N = H3 A/Brisbane/10/2007 (points overlap). EI07: modest rise against W = H3 A/Wisconsin/67/2005, N = H3 A/Brisbane/10/2007, J = H3 A/Johannesburg/33/1994, and H = H1 A/California/04/2009 (J and H points overlap). EI12: rise against W = H3 A/Wisconsin/67/2005 and N = H3 A/Brisbane/10/2007 (points overlap). EI13: modest rise against W = H3 A/Wisconsin/67/2005 (13.7-fold) and N = H3 A/Brisbane/10/2007 (14.7-fold) (points overlap).
(PDF)

Figure S2 **Plasmacytosis following TIV or EI.** Plots shown are of total B cell populations (CD3/14/16/235a^−^ CD19^+^) and are normalized to 10000 events per panel. Ellipse in upper right corner of each panel is homologous to the sorting gate used for the isolation of single plasma cells for mAb generation. Additional gating on CD20 was also performed for cell sorting and the final population sorted for mAb production was CD3/14/16/235a^−^ CD19^+^ CD20^−/lo^ CD27^hi^ CD38^hi^. Percentages shown are of plasma cells as a fraction of total B cells.
(PDF)

Figure S3 **Reactivities of human rmAbs recovered from TIV and EI.** A. Antibodies from TIV subjects. We recovered plasma cells producing rmAbs against influenza antigens from all TIV subjects. In two subjects (TIV01 and TIV21) the majority of recovered rmAbs were reactive with influenza [179/245 (73%) and 39/41 (95%), respectively]. In the other three subjects, less than half of recovered rmAbs were influenza-specific [TIV04 8/17 (47%), TIV14 1/16 (6%), TIV24 35/85 (41%)]. B. Antibodies from EI subjects. We recovered plasma cells producing rmAbs against influenza antigens from five of six EI subjects; from one subject (EI12) we recovered 51 rmAbs that were not reactive for any antigen tested. None of the other EI subjects had more than 25% of rmAbs reactive with influenza [EI02 4/31 (13%), EI03 11/108 (10%), EI05 3/141 (2.1%), EI07 3/34 (9%), EI13 18/86 (21%)].
(PDF)

Figure S4 **Relationship between influenza reactivity and clonal lineages from TIV and EI subjects.** Influenza-specific rmAbs recovered from TIV were more likely to be in a clonal lineage compared with EI. In TIV, 93/404 (23%) of rmAbs were influenza-specific but not a member of a clonal lineage (blue wedge), 159/404 (39%) were both influenza-specific and members of clonal lineages (purple wedge), while only 16/404 (4%) were members of clonal lineages but not reactive with influenza antigens (red wedge). In EI, 31/451 (6.9%) were influenza-specific but not part of a clonal lineage, 6/451 (1.3%) were both influenza-specific and part of a clonal lineage, and 22/451 (4.9%) were not influenza specific but were part of a clonal lineage.
(PDF)

Figure S5 **VDJ mutation rate of influenza-specific mAbs from TIV and EI subjects.** VDJ mutation rates in rmAbs from TIV subjects (range 0.9–30.3%, mean 5.8%±0.2%) were lower on average than rmAbs from EI subjects (range 0.4–15.8%, mean 8.6%±0.6%) (two-tailed *t*-test, *p*<0.0001).
(PDF)

Figure S6 **VDJ mutation rate of influenza-specific mAbs from TIV and EI subjects by isotype of mAb.** A. HC isotype of influenza-specific rmAbs from TIV subjects were found to be predominantly IgG1 (201/252, 80%), followed by IgA1 (36/252, 14%) and IgM (9/252, 3.6%). VDJ mutation rates for these three isotypes were as follows: IgG1 5.9%±0.2%, IgA1 6.1%±0.8%, IgM 3.9%±0.4%. Comparison of mutation rates between isotypes within the TIV group did not show any significant differences. B. HC isotype of influenza-specific rmAbs from EI subjects were also found to be predominantly IgG1 (23/37, 62%), followed by IgA1 (7/37, 19%) and IgM (5/37, 14%). VDJ mutation rates for these three isotypes were as follows: IgG1 9.1%±0.7%, IgA1 7.2%±1.7%, IgM 8.2%±2.1%. Comparison of mutation rates between isotypes within the EI group did not show any significant differences. When compared between the TIV and EI groups, however, mutation rates for both IgG1 and IgM were found to be higher in rmAbs derived from EI subjects vs. those derived from TIV subjects (two-tailed *t*-test, *p*<0.0001 for IgG1, *p* = 0.033 for IgM).
(PDF)

Figure S7 **HC CDR3 length distribution of mAbs from TIV and EI subjects.** A. HC CDR3 length distribution of all mAbs isolated from TIV subjects showed a predominance of mAbs with length 19; as with influenza-specific mAbs (Fig. S7C online) a large portion of this was contributed by 13 clonal lineages from one subject (TIV01) that had similar heavy chain rearrangements (V_H_4-59–J_H_6) but that did not share light chains. These clonal lineages contributed 68 mAbs (gray portion of bar). B. HC CDR3 length distribution of all mAbs isolated from EI subjects showed a distribution similar to that of the influenza-specific rmAbs (Fig. S7D online). As with influenza-specific mAbs, HC CDR3 lengths of 16 were the most common overall. Kolmogorov-Smirnov test of the distributions in A and B showed a difference in distribution (test statistic = 4.67, *p*<0.0001). C. HC CDR3 length distribution of influenza-specific rmAbs from TIV subjects. The number of aas in HC CDR3 was most commonly 19; 13 clonal lineages from subject TIV01 with similar rearrangements (V_H_4-59–J_H_6) contributed 65 rmAbs to this peak (gray portion of bar). D. HC CDR3 length distribution of influenza-specific rmAbs from EI subjects. HC CDR3 lengths of 16 were most common. Kolmogorov-Smirnov test of the distributions in C and D showed a difference in distribution (test statistic = 2.25, *p*<0.0001).
(PDF)

Figure S8 **Distribution of rmAb reactivities among influenza-specific rmAbs from TIV and EI subjects.** For each tested antigen, influenza-specific rmAbs were counted to determine how many rmAbs reacted with that antigen. In contrast to the other analyses, each reactivity was counted separately (*e.g.*, rmAb reactive with 2007–2008 influenza vaccine and with H3 A/Wisconsin/67/2005 was counted as positive for both columns). Multiply reactive rmAbs were counted as positive for each antigen with which they reacted; bars do not sum to 100% for this analysis. For each TIV subject, rmAb reactivity was found to be primarily specific for individual strains. TIV01 (173 influenza-specific rmAbs): 171/173 (98.9%) reacted with 2007–2008 influenza vaccine, 149/173 (86%) reacted with H1 A/Solomon Islands/03/2006 while only 1/173 (0.6%) reacted with H3 A/Wisconsin/67/2005. SPR testing of rmAbs derived from subject TIV01 bound to rHA H1 A/Solomon Islands/03/2006 but not to H3 A/Wisconsin/67/2005 (Figs. S9A, S9B, S9C online). TIV04 (8 influenza-specific rmAbs): 8/8 (100%) reacted with 2007–2008 influenza vaccine, 2/8 (25%) reacted with H1 A/Solomon Islands/03/2006; no other reactivities detected. TIV14 omitted from this analysis as there was only one influenza-specific rmAb isolated (this rmAb reacted with 2007–2008 influenza vaccine, H1 A/Solomon Islands/03/2006, and H1 A/Brisbane/59/2007). TIV21 (39 influenza-specific rmAbs): 39/39 (100%) reacted with 2008–2009 influenza vaccine, 28/39 (72%) reacted with HA B/Florida/04/2006, while only 5/39 (13%) reacted with H1 A/Brisbane/59/2007 and 2/39 (5%) reacted with H3 A/Brisbane/10/2007. TIV24 (31 influenza-specific rmAbs): 26/31 (84%) reacted with 2008–2009 influenza vaccine, 18/31 (58%) reacted with H1 A/Brisbane/59/2007, while only 2/31 (6%) reacted with H3 A/Brisbane/10/2007 and 1/31 (3%) reacted with HA B/Florida/04/2006. Additionally, 18/31 (58%) reacted with H1 A/Solomon Islands/03/2006, however, only 4/31 (13%) reacted with 2007–2008 influenza vaccine. For EI subjects, rmAbs were more commonly cross-reactive/less dominantly directed against single influenza strains. EI02 (4 influenza-specific rmAbs): 3/4 (75%) reacted with 2007–2008 influenza vaccine, 2/4 (50%) reacted with H3 A/Wisconsin/67/2005, 2/4 (50%) reacted with H3 A/Brisbane/10/2007, and 3/4 (75%) reacted with H3 A/Johannesburg/33/1994. No rmAbs reacted with H1, H5, or B strain HAs. EI03 (10 influenza-specific rmAbs): 4/10 (40%) reacted with 2007–2008 influenza vaccine, 3/10 (30%) rmAbs reacted with 2008–2009 influenza vaccine, 4/10 (40%) reacted with H3 A/Wisconsin/67/2005, 5/10 (50%) reacted with H3 A/Brisbane/10/2007, and 8/10 (80%) reacted with H3 A/Johannesburg/33/1994. Additionally, 1/10 (10%) reacted with H1 A/California/04/2009; no binding was found to other H1, H5, or B strain HAs. EI05 (3 influenza-specific rmAbs): 1/3 (33%) reacted with 2007–2008 influenza vaccine, 2/3 (67%) reacted with H3 A/Wisconsin/67/2005 No binding was found to other H1, H3, H5, or B strain HAs. EI07 (3 influenza-specific rmAbs): 1/3 (33%) reacted with 2007–2008 influenza vaccine, 1/3 (33%) rmAbs reacted with 2008–2009 influenza vaccine, 2/3 (67%) reacted with H3 A/Wisconsin/67/2005, 1/3 (33%) reacted with H3 A/Brisbane/10/2007, and 2/3 (67%) reacted with H3 A/Johannesburg/33/1994. No binding was found to other H1, H5, or B strain HAs. EI12 was omitted as there were no influenza-specific rmAbs isolated. EI13 (17 influenza-specific rmAbs): 8/17 (47%) reacted with 2007–2008 influenza vaccine, 8/17 (47%) rmAbs reacted with 2008–2009 influenza vaccine, 7/17 (41%) reacted with H3 A/Wisconsin/67/2005, 9/17 (53%) reacted with H3 A/Brisbane/10/2007, and 9/17 (53%) reacted with H3 A/Johannesburg/33/1994. Additionally, 1/17 (6%) reacted with H1 A/Solomon Islands/03/2006, 2/17 (12%) reacted with H1 A/Brisbane/59/2007, 1/17 (6%) reacted with H1 A/California/04/2009, and 4/17 (24%) reacted with B/Florida/04/2006. No rmAbs reacted with H5 HAs. SPR testing of selected rmAbs from EI13 showed binding to rHAs from both H1 A/Solomon Islands/03/2006 and H3 A/Wisconsin/67/2005 (Figs. S9B, S9C online).
(PDF)

Figure S9 **Surface plasmon resonance analysis of rmAbs recovered from TIV and EI subjects.** A. Human rmAbs 1248, 1258, and 1270 from subject TIV01 bound to H1 A/Solomon Islands/03/2006 bound to an SPR chip. B. Human rmAbs 1210 and 1267 from subject TIV01 and rmAbs 2210 and 2217 from subject EI13 bound to H1 A/Solomon Islands/03/2006 bound to an SPR chip. C. Human rmAbs 1267 and 1210 from subject TIV01 did not bind to H3 A/Wisconsin/67/2005 bound to an SPR chip while rmAbs 2210 and 2217 from subject EI13 did bind. D. Human rmAbs from subjects TIV01 (1210 and 1267) and EI13 (2210 and 2217) showed essentially no interaction with phosphatidylcholine-cardiolipin liposomes. Anti-HIV-1 mAb 4E10 is shown as a positive control and anti-HIV-1 mAbs 2F5 and 13H11 are shown as negative controls. E. Human rmAb 1258 from subject TIV01 shows some degree of binding to phosphatidylcholine-cardiolipin liposomes while rmAb 1270 from subject TIV01 shows a lesser degree of binding. Anti-HIV-1 mAb 4E10 is shown as a positive control. No binding of rmAbs to apoferritin (control protein) or to phosphatidylcholine-phosphatidylserine liposomes was seen (data not shown).
(PDF)

Figure S10 **Additional representative clonal lineage of rmAbs from subject TIV01.** Data for starred rmAbs appear in Table S12 online. Clonal lineage 643 from subject TIV01. Sixteen of 17 rmAbs (94%) bound one antigen, 1/17 (6%) was not influenza-specific. Four rmAbs were tested in additional assays. Affinity for rHA binding was measured for three rmAbs and all had sub-nanomolar affinity for rHA H1 A/Solomon Islands/03/2006 and no binding to H3 A/Wisconsin/67/2005. Neutralization assays for one rmAb (1267 IgG1) showed potent neutralization of H1N1 A/Solomon Islands/03/2006, weak neutralization of H1N1 A/Brisbane/59/2007 and no neutralization of H3N2 A/Wisconsin/67/2005. Two other rmAbs were tested only against H1N1 A/Brisbane/59/2007 and neither neutralized. All four tested rmAbs showed potent HAI against H1N1 A/Solomon Islands/03/2006; two rmAbs showed weak HAI activity against H3N2 A/Wisconsin/67/2005.
(PDF)

Figure S11 **Additional representative clonal lineages of rmAbs from TIV subjects.** A. Clonal lineage 690 from subject TIV01. Three of 12 rmAbs (25%) bound one antigen, 8/12 (67%) bound two antigens, 1/12 (8%) bound none. B. Clonal lineage 2737 from subject TIV21. Five of 8 rmAbs (63%) bound one antigen, 3/8 (37%) bound two.
(PDF)

Figure S12 **Sequence alignment of H3 HAs used in this study.** Amino acid sequences for H3 HA strains used in this study were downloaded from PubMed and aligned to the H3 A/Wisconsin/67/2005 sequence. Differences are highlighted in colors for each aligned sequence. The H3 A/Johannesburg/33/1994 strain was the least similar with 88.8% identity. Only the HA1 sequence for the Johannesburg strain was available; all sequences were aligned in this region only.
(PDF)

Table S1 **Clonal lineages of antibodies from TIV subjects.**
(PDF)

Table S2 **Clonal lineages of antibodies from EI subjects.**
(PDF)

Table S3 **Isotypes of isolated influenza-specific rmAbs.**
(PDF)

Table S4 **Isotypes of isolated rmAbs not specific for influenza antigens.**
(PDF)

Table S5 **Heavy chain family usage of influenza-specific rmAbs.**
(PDF)

Table S6 **Heavy chain family usage of isolated rmAbs not specific for influenza antigens.**
(PDF)

Table S7 **Kappa chain family usage of isolated influenza-specific rmAbs.**
(PDF)

Table S8 **Kappa chain family usage of isolated rmAbs not specific for influenza antigens.**
(PDF)

Table S9 **Lambda chain family usage of isolated influenza-specific rmAbs.**
(PDF)

Table S10 **Lambda chain family usage of isolated rmAbs not specific for influenza antigens.**
(PDF)

Table S11 **Characteristics of cross-reactive rmAbs.**
(PDF)

## References

[1] Thompson WW, Shay DK, Weintraub E, Brammer L, Bridges CB (2004). Influenza-associated hospitalizations in the United States.. JAMA.

[2] Thompson WW, Shay DK, Weintraub E, Brammer L, Cox N (2003). Mortality associated with influenza and respiratory syncytial virus in the United States.. JAMA.

[3] Anonymous (2009). Update: influenza activity–United States, April-August 2009.. MMWR Morb Mortal Wkly Rep.

[4] Johnson NP, Mueller J (2002). Updating the accounts: global mortality of the 1918–1920 “Spanish” influenza pandemic.. Bull Hist Med.

[5] Fiore AE, Shay DK, Broder K, Iskander JK, Uyeki TM (2009). Prevention and control of seasonal influenza with vaccines: recommendations of the Advisory Committee on Immunization Practices (ACIP), 2009.. MMWR Recomm Rep.

[6] Wrammert J, Smith K, Miller J, Langley WA, Kokko K (2008). Rapid cloning of high-affinity human monoclonal antibodies against influenza virus.. Nature.

[7] Knossow M, Skehel JJ (2006). Variation and infectivity neutralization in influenza.. Immunology.

[8] Karlsson Hedestam GB, Fouchier RA, Phogat S, Burton DR, Sodroski J (2008). The challenges of eliciting neutralizing antibodies to HIV-1 and to influenza virus.. Nat Rev Microbiol.

[9] Ekiert DC, Bhabha G, Elsliger MA, Friesen RH, Jongeneelen M (2009). Antibody recognition of a highly conserved influenza virus epitope.. Science.

[10] Kashyap AK, Steel J, Oner AF, Dillon MA, Swale RE (2008). Combinatorial antibody libraries from survivors of the Turkish H5N1 avian influenza outbreak reveal virus neutralization strategies.. Proc Natl Acad Sci U S A.

[11] Sui J, Hwang WC, Perez S, Wei G, Aird D (2009). Structural and functional bases for broad-spectrum neutralization of avian and human influenza A viruses.. Nat Struct Mol Biol.

[12] Corti D, Suguitan AL, Pinna D, Silacci C, Fernandez-Rodriguez BM (2010). Heterosubtypic neutralizing antibodies are produced by individuals immunized with a seasonal influenza vaccine.. J Clin Invest.

[13] Zaas AK, Chen M, Varkey J, Veldman T, Hero AO (2009). Gene expression signatures diagnose influenza and other symptomatic respiratory viral infections in humans.. Cell Host Microbe.

[14] Liao HX, Levesque MC, Nagel A, Dixon A, Zhang R (2009). High-throughput isolation of immunoglobulin genes from single human B cells and expression as monoclonal antibodies.. J Virol Methods.

[15] Renfrey S, Watts A (1994). Morphological and biochemical characterization of influenza vaccines commercially available in the United Kingdom.. Vaccine.

[16] Davenport FM, Hennessy AV, Francis T (1953). Epidemiologic and immunologic significance of age distribution of antibody to antigenic variants of influenza virus.. The Journal of experimental medicine.

[17] Fazekas de St G, Webster RG (1966). Disquisitions on Original Antigenic Sin. II. Proof in lower creatures.. J Exp Med.

[18] Fazekas de St G, Webster RG (1966). Disquisitions of Original Antigenic Sin. I. Evidence in man.. J Exp Med.

[19] Webster RG (1966). Original antigenic sin in ferrets: the response to sequential infections with influenza viruses.. J Immunol.

[20] Wrammert J, Koutsonanos D, Li GM, Edupuganti S, Sui J (2011). Broadly cross-reactive antibodies dominate the human B cell response against 2009 pandemic H1N1 influenza virus infection.. J Exp Med.

[21] Yu X, Tsibane T, McGraw PA, House FS, Keefer CJ (2008). Neutralizing antibodies derived from the B cells of 1918 influenza pandemic survivors.. Nature.

[22] Kashyap AK, Steel J, Rubrum A, Estelles A, Briante R (2010). Protection from the 2009 H1N1 pandemic influenza by an antibody from combinatorial survivor-based libraries.. PLoS Pathog.

[23] Forgie SE, Keenliside J, Wilkinson C, Webby R, Lu P (2011). Swine outbreak of pandemic influenza A virus on a Canadian research farm supports human-to-swine transmission.. Clinical infectious diseases : an official publication of the Infectious Diseases Society of America.

[24] Lau LL, Cowling BJ, Fang VJ, Chan KH, Lau EH (2010). Viral shedding and clinical illness in naturally acquired influenza virus infections.. The Journal of Infectious Diseases.

[25] Katz JM, Lim W, Bridges CB, Rowe T, Hu-Primmer J (1999). Antibody response in individuals infected with avian influenza A (H5N1) viruses and detection of anti-H5 antibody among household and social contacts.. The Journal of Infectious Diseases.

[26] Anonymous (2009). Update on influenza A (H1N1) 2009 monovalent vaccines.. MMWR Morb Mortal Wkly Rep.

[27] Whittle J, Zhang R, Khurana S, King LR, Manischewitz J (2011). Broadly neutralizing human antibody that recognizes the receptor-binding pocket of influenza virus hemagglutinin.. Proc Natl Acad Sci U S A.

[28] De Filette M, Min Jou W, Birkett A, Lyons K, Schultz B (2005). Universal influenza A vaccine: optimization of M2-based constructs.. Virology.

[29] Schotsaert M, De Filette M, Fiers W, Saelens X (2009). Universal M2 ectodomain-based influenza A vaccines: preclinical and clinical developments.. Expert Rev Vaccines.

[30] Kwong PD, Wilson IA (2009). HIV-1 and influenza antibodies: seeing antigens in new ways.. Nat Immunol.

[31] Wei CJ, Boyington JC, McTamney PM, Kong WP, Pearce MB (2010). Induction of broadly neutralizing H1N1 influenza antibodies by vaccination.. Science.

[32] Khurana S, Chearwae W, Castellino F, Manischewitz J, King LR (2010). Vaccines with MF59 adjuvant expand the antibody repertoire to target protective sites of pandemic avian H5N1 influenza virus.. Sci Transl Med.

[33] McKean D, Huppi K, Bell M, Staudt L, Gerhard W (1984). Generation of antibody diversity in the immune response of BALB/c mice to influenza virus hemagglutinin.. Proc Natl Acad Sci U S A.

[34] Clarke SH, Huppi K, Ruezinsky D, Staudt L, Gerhard W (1985). Inter- and intraclonal diversity in the antibody response to influenza hemagglutinin.. J Exp Med.

[35] Clarke S, Rickert R, Wloch MK, Staudt L, Gerhard W (1990). The BALB/c secondary response to the Sb site of influenza virus hemagglutinin. Nonrandom silent mutation and unequal numbers of V_H_ and V_k_ mutations.. J Immunol.

[36] Sangster MY, Topham DJ, D'Costa S, Cardin RD, Marion TN (2000). Analysis of the virus-specific and nonspecific B cell response to a persistent B-lymphotropic gammaherpesvirus.. J Immunol.

[37] Krause JC, Tumpey TM, Huffman CJ, McGraw PA, Pearce MB (2010). Naturally occurring human monoclonal antibodies neutralize both 1918 and 2009 pandemic influenza A (H1N1) viruses.. J Virol.

[38] Clarke SH, Staudt LM, Kavaler J, Schwartz D, Gerhard WU (1990). V region gene usage and somatic mutation in the primary and secondary responses to influenza virus hemagglutinin.. J Immunol.

[39] Throsby M, van den Brink E, Jongeneelen M, Poon LL, Alard P (2008). Heterosubtypic neutralizing monoclonal antibodies cross-protective against H5N1 and H1N1 recovered from human IgM^+^ memory B cells.. PLoS One.

[40] Sasso EH, Johnson T, Kipps TJ (1996). Expression of the immunoglobulin VH gene 51p1 is proportional to its germline gene copy number.. J Clin Invest.

[41] Pape KA, Taylor JJ, Maul RW, Gearhart PJ, Jenkins MK (2011). Different B cell populations mediate early and late memory during an endogenous immune response.. Science.

[42] Wang TT, Tan GS, Hai R, Pica N, Petersen E (2010). Broadly protective monoclonal antibodies against H3 influenza viruses following sequential immunization with different hemagglutinins.. PLoS Pathog.

[43] Laurie KL, Carolan LA, Middleton D, Lowther S, Kelso A (2010). Multiple infections with seasonal influenza A virus induce cross-protective immunity against A(H1N1) pandemic influenza virus in a ferret model.. J Infect Dis.

[44] Wang TT, Tan GS, Hai R, Pica N, Ngai L (2010). Vaccination with a synthetic peptide from the influenza virus hemagglutinin provides protection against distinct viral subtypes.. Proc Natl Acad Sci U S A.

[45] Steel J, Lowen AC, Wang T, Yondola M, Gao Q (2010). Influenza virus vaccine based on the conserved hemagglutinin stalk domain.. MBio.

[46] Dal Porto JM, Haberman AM, Kelsoe G, Shlomchik MJ (2002). Very low affinity B cells form germinal centers, become memory B cells, and participate in secondary immune responses when higher affinity competition is reduced.. J Exp Med.

[47] Shih TA, Meffre E, Roederer M, Nussenzweig MC (2002). Role of BCR affinity in T cell dependent antibody responses in vivo.. Nat Immunol.

[48] Jackson GG, Dowling HF, Spiesman IG, Boand AV (1958). Transmission of the common cold to volunteers under controlled conditions. I. The common cold as a clinical entity.. AMA Arch Intern Med.

[49] Smith K, Garman L, Wrammert J, Zheng NY, Capra JD (2009). Rapid generation of fully human monoclonal antibodies specific to a vaccinating antigen.. Nat Protoc.

[50] Ewing B, Green P (1998). Base-calling of automated sequencer traces using phred. II. Error probabilities.. Genome Res.

[51] Ewing B, Hillier L, Wendl MC, Green P (1998). Base-calling of automated sequencer traces using phred. I. Accuracy assessment.. Genome Res.

[52] Kepler TB, Sample C, Hudak K, Roach J, Haines A (2010). Chiropteran types I and II interferon genes inferred from genome sequencing traces by a statistical gene-family assembler.. BMC Genomics.

[53] Smith TF, Waterman MS (1981). Identification of common molecular subsequences.. J Mol Biol.

[54] Volpe JM, Cowell LG, Kepler TB (2006). SoDA: implementation of a 3D alignment algorithm for inference of antigen receptor recombinations.. Bioinformatics.

[55] Felsenstein J (2005). PHYLIP (Phylogeny Inference Package). 3.6 ed.

[56] Gray ES, Taylor N, Wycuff D, Moore PL, Tomaras GD (2009). Antibody specificities associated with neutralization breadth in plasma from human immunodeficiency virus type 1 subtype C-infected blood donors.. J Virol.

[57] Alam SM, Scearce RM, Parks RJ, Plonk K, Plonk SG (2008). Human immunodeficiency virus type 1 gp41 antibodies that mask membrane proximal region epitopes: antibody binding kinetics, induction, and potential for regulation in acute infection.. J Virol.

[58] Tomaras GD, Yates NL, Liu P, Qin L, Fouda GG (2008). Initial B-cell responses to transmitted human immunodeficiency virus type 1: virion-binding immunoglobulin M (IgM) and IgG antibodies followed by plasma anti-gp41 antibodies with ineffective control of initial viremia.. J Virol.

[59] Alam SM, McAdams M, Boren D, Rak M, Scearce RM (2007). The role of antibody polyspecificity and lipid reactivity in binding of broadly neutralizing anti-HIV-1 envelope human monoclonal antibodies 2F5 and 4E10 to glycoprotein 41 membrane proximal envelope epitopes.. J Immunol.

[60] Cottey R, Rowe CA, Bender BS (2001). Influenza virus.. Curr Protoc Immunol Chapter.

