# Supplementary material for: H3N2 Influenza Infection Elicits More Cross-Reactive and Less Clonally Expanded Anti-Hemagglutinin Antibodies Than Influenza Vaccination
Source: PLoS One. 2011 Oct 19;6(10):e25797. doi: 10.1371/journal.pone.0025797 (PMC3198447; doi:10.1371/journal.pone.0025797)
Supplement: Text S1 — Additional description of results presented in Supplemental Information. (PDF) [file pone.0025797.s001.pdf]

Supplemental Text (1384 words)

TIV rmAb Reactivity Similar to Plasma Antibody Repertoire. The antibody response in TIV subjects was restricted to the subtypes present in the administered vaccine (Figs. S1, S8 online). Following TIV, subject TIV01 had a rise in plasma antibody titer against rHA H1 A/Solomon Islands/03/2006 (Fig. S1 online) and the great majority of rmAbs isolated from this subject (149/173; 86%) were also reactive with that rHA (Fig. S8 online). Antibodies from subject TIV04 showed a pattern similar to TIV01. Subject TIV21 had a rise in antibody titer against rHA B/Florida/04/2006 (Fig. S1 online) and the majority of isolated rmAbs (28/39, 72%) reacted with that rHA (Fig. S8 online). Subject TIV24 showed a rise in antibody titers against rHA H1 A/Brisbane/59/2007 and rHA H1 A/Solomon Islands/03/2006 (Fig. S1 online); isolated rmAbs paralleled this specificity with 18/31 (58%) and 18/31 (58%) reacting with those two rHAs, respectively (Fig. S8 online).

In contrast, the strong correlation between specific rHA-binding antibody titers and the isolation of corresponding rmAbs was not seen in EI subjects (Fig. S1, Fig S8 online). For example, subject EI13 generated plasma cells from which the most broadly cross-reactive rmAbs were recovered (Fig. 3; Fig. S8, Table S11 online) but 28 days after infection exhibited only modest increases in rHA binding [13.7-fold increase in titer against H3 A/Wisconsin/67/2005; Fig. S1 online] and virus neutralization [2-fold increase; Table 1]. These data suggested that the broad neutralizing clonal lineage isolated from EI13 (Fig. 3) did not contribute significantly to plasma antibody 28 days following EI.

Antibody Isotype, and HC and LC Gene Family Usage in TIV and EI. The isotype of influenza-specific rmAbs from both TIV and EI was predominantly IgG1 [201/252 (80%) and 23/37 (62%), respectively] (Table S3, Fig. S5 online). The next most common isotypes of influenza-specific rmAbs were IgA1 [TIV 36/252 (14%), EI 7/37 (19%)] and IgM [TIV 9/252 (4%), EI 5/37 (14%)] (Table S3 online). Antibodies that did not bind influenza antigens were also primarily of these three isotypes, although the patterns were different between TIV and EI (IgG1 > IgA1 > IgM in TIV; IgA1 > IgM > IgG1 in EI) (Table S4 online).

Using the SoDA inference algorithm (1), we analyzed V(D)J sequences from all the mAbs isolated in this study. For rmAbs not reactive with influenza antigens, HC family gene usage was similar between TIV and EI subjects (Table S6 online). In contrast, influenza-specific rmAbs from TIV subjects used primarily V_H3 (108/252, 43%) and V_H4 (124/252, 49%) while influenza-specific rmAbs from EI subjects used primarily V_H1 and V_H3 (Table S5 online). The predominance of V_H4 usage in the TIV antibodies included a large number of mAbs from one subject (TIV01) who had 13 clonal lineages with similar IgH rearrangements (V_H4-59–J_H6) but dissimilar LC rearrangements. The difference between heavy chain usage between TIV and EI subjects seen here likely reflects the greater degree of clonal expansion in mAbs isolated from TIV subjects.

More than half of influenza-specific rmAbs from both TIV and EI subjects used kappa chains [145/252 (58%) and 28/37 (76%), respectively] (Table S7 online) and this kappa chain preference was greater in EI subjects compared to TIV subjects ($\chi^2 = 4.4$, $p = 0.036$). In mAbs from both TIV and EI subjects, $V_\kappa1$ was the most used V-gene family

[TIV 103/145 (71%); EI 15/28 (54%)] (Table S7 online). In mAbs from TIV subjects, this $V_{\kappa}1$ bias reflected nine clonal lineages from subject TIV01; 9/13 clonal lineages (52 influenza-specific rmAbs) expressing V_H4-59–J_H6 were associated with $V_{\kappa}1$ bearing LCs (Table S1 online). However, $V_{\kappa}1$ rearrangements were also over represented in rmAbs from TIV and EI subjects not reactive with influenza [TIV 65/105 (62%); EI 140/291 (48%)] (Table S8 online). Of antigen-specific rmAbs using lambda chains, $V_{\lambda}1$ was most common [TIV 51/107 (48%); EI 8/9 (89%)] (Table S9 online), while in rmAbs not specific for influenza, a wider distribution of V_{λ} family usage was observed (Table S10 online).

Higher VDJ Mutation Rates in EI Subjects. Using the SoDA inference algorithm (1), we analyzed HC and LC sequences from all 855 mAbs isolated. Influenza-specific rmAbs from EI had a higher VDJ mutation frequency than rmAbs from TIV (TIV range 0.9-30.3%, mean 5.8% ± 0.2%; EI range 0.4-15.8%, mean 8.6% ± 0.6%; two-tailed *t*-test, $p <$ 0.0001) (Fig. S5 online). Of IgM mAbs isolated, there was a higher frequency of mutation seen in EI compared to TIV (8.2% ± 2.1% in 5 mAbs vs. 3.9% ± 0.4% in 9 mAbs, respectively, two-tailed *t*-test, $p = 0.033$) (Fig. S6 online). In addition, 3/5 IgM mAbs from EI were members of clonal lineage 2569 that included one IgA1 mAb (Fig. 3).

HCDR3 Lengths Are Consistent with the Higher Degree of Clonal Expansion Observed in TIV Compared with EI. We analyzed the distribution of HCDR3 lengths in all mAbs isolated from each of the study groups and found that they were different (Kolmogorov-

Smirnov test statistic = 4.67, $p < 0.0001$) (Figs. S7A, S7B online). We also analyzed the distribution of influenza-specific mAbs isolated from TIV and EI subjects and found that they were different (Kolmogorov-Smirnov test statistic = 2.25, $p < 0.0001$) (Figs. S7C, S7D online). Within the distribution of mAbs from TIV subjects there were 115 total HC and 94 influenza-specific HC with HCDR3s of 19 aas [range 6-25 aas, peak 19 aas] (Figs. S7A, S7C online). As was seen in the analysis of HC family usage in TIV subjects, a large number of 19 aa HCDR3s derived from one subject (TIV01) from 13 clonal lineages with similar HC rearrangements (V_H4-59–J_H6). These clonal lineages contributed 68 mAbs of which 65 were influenza-specific, and these mAbs altered the distribution in influenza-reactive mAbs from TIV subjects (Fig. S7C online) and in the overall population of isolated mAbs [range 6-27 aas, peak 19 aas] (Fig. S7A online). HCDR3 lengths in influenza-specific rmAbs derived from EI subjects did not show a similar peak at 19 aas [range 9-25 aas, peak 16 aas] (Fig. S7D online); neither did HCDR3 lengths from all isolated mAbs from EI subjects [range 6-27 aas, peak 16 aas] (Fig. S7B online). The peak at 19 aas in mAbs derived from TIV subjects reflects the degree of clonal expansion in this subject group.

Additional Clonal Lineages from TIV Subjects. Lineage 643 contains 16/17 (94%) members reactive with rHA H1 A/Solomon Islands/03/2006 (Fig. S10 online). Affinity measurements of three members (1210, 1248, and 1267) show small differences among rHA affinities (Fig. S10 online), consistent with their close phylogenetic relationship. Neutralization and HAI testing also demonstrate specificity for H1N1 A/Solomon

Islands/03/2006 with minimal cross-reactivity seen to other H1N1 or H3N2 isolates (Fig. S10 online).

Lineage 690 shows evidence of greater sequence diversity than lineages 641 or 643 and a greater degree of antibody binding diversity (Fig. S11A online). Three lineage members bound only rHA H1 A/Solomon Islands/03/2006 while 8/12 (67%) of lineage members bound two rHAs (H1 A/Solomon Islands/03/2006 and H1 A/Brisbane/59/2007). Interestingly, the dendrogram branch length (and by inference degree of mutation from the consensus sequence for the dendrogram) for any single mAb did not correlate with increased binding, as rmAb 1275 had a greater degree of sequence divergence compared to other lineage members and was less reactive than its nearest neighbor (rmAb 1312). Similarly, rmAb 1324 did not react with any antigen tested while its nearest neighbor (rmAb 1283) reacted with two rHAs. As with lineage 641, this is consistent with a random process of Ig gene mutation being observed at a single time point in the plasma cell repertoire in subject TIV01.

Lineage 2737 from subject TIV21 is composed entirely of IgA1 members of which eight were reactive with rHA B/Florida/04/2006 and three members also reactive with TIV 2007-2008 (Fig. S11B online). This additional reactivity was present in three distinct branches of the dendrogram and suggested that no single change was responsible for the added reactivity.

HA Sequence Homology Among H3 Isolates Tested. Amino acid sequences for H3 HA isolates used in this study were downloaded from PubMed and aligned to the H3 A/Wisconsin/67/2005 strain (Fig. S12 online). The H3 A/Johannesburg/33/1994 strain

was the least similar (88.8% identity) while other sequences were more similar (95.8-98.3% sequence identity). Isolation of rmAbs from EI subjects that were reactive only with the most dissimilar HA sequence suggested the recall of a previously stimulated population of B cells.

References

1. Volpe, J.M., Cowell, L.G., and Kepler, T.B. 2006. SoDA: implementation of a 3D alignment algorithm for inference of antigen receptor recombinations. *Bioinformatics* 22:438-444.
