# Supplementary figures and images for: H3N2 Influenza Infection Elicits More Cross-Reactive and Less Clonally Expanded Anti-Hemagglutinin Antibodies Than Influenza Vaccination
Source: PLoS One. 2011 Oct 19;6(10):e25797. doi: 10.1371/journal.pone.0025797 (PMC3198447; doi:10.1371/journal.pone.0025797)

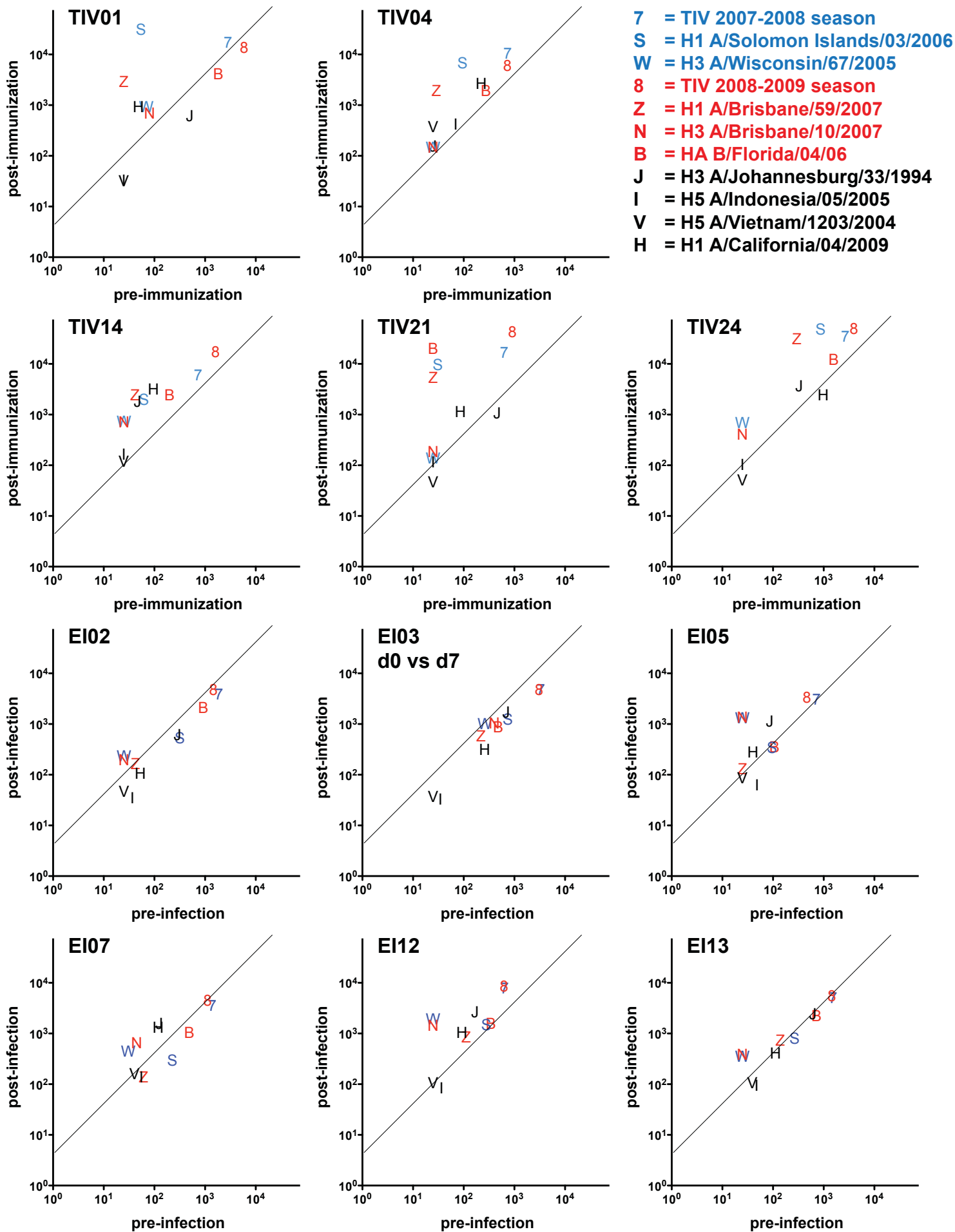

**Figure S1**

Supplement: Figure S1 — Influenza antigen binding titers of plasma from TIV and EI subjects. Plasma samples from day 0 and from day 21 (TIV) or day 28 (EI) were tested by ELISA for binding to split virus vaccine preparations and to purified recombinant hemagglutinins. For subject EI03, no day 28 plasma sample was available; a day 7 plasma sample was substituted for this analysis. ELISA was performed using serial dilutions and optical density readings were fitted to a 5-parameter curve; endpoint titers were determined as three fold over the background of the assay for each run. Data are plotted as reciprocal titer values and are pre-immunization/pre-infection titer (x-axis) vs. post-immunization/post-infection titer (y-axis). The threshold of the assay was 1∶25 dilution; endpoint titers that fell below that cutoff were adjusted to that value. The diagonal line for each plot represents a four-fold rise in titer; distance above the diagonal line is proportional to boosting. Each antigen is represented by a number or letter; these are color coded (blue for antigens contained in the 2007–2008 vaccine, red for antigens in the 2008–2009 vaccine, black for antigens unrelated to either vaccine). The code for the graphs is as follows: 7 = 2007–2008 influenza vaccine (blue); S = H1 A/Solomon Islands/03/2006 (blue); W = H3 A/Wisconsin/67/2005 (blue); 8 = 2008–2009 influenza vaccine (red); Z = H1 A/Brisbane/59/2007 (red); N = H3 A/Brisbane/10/2007 (red); B = B/Florida/04/2006 (red); J = H3 A/Johannesburg/33/1994 (black); I = H5 A/Indonesia/05/2005 (black); V = H5 A/Vietnam/1203/2004 (black); H = H1 A/California/04/2009 (black). For individual subjects, reactivity to specific strains was found to dominate. TIV01: rise against S = H1 A/Solomon Islands/03/2006. TIV04: rise against S = H1 A/Solomon Islands/03/2006. TIV14: No clear dominant response. TIV21: rise against 8 = 2008–2009 influenza vaccine and B = B/Florida/04/2006. TIV24: rise against 8 = 2008–2009 influenza vaccine and Z = H1 A/Brisbane/59/200 [file pone.0025797.s002.pdf]

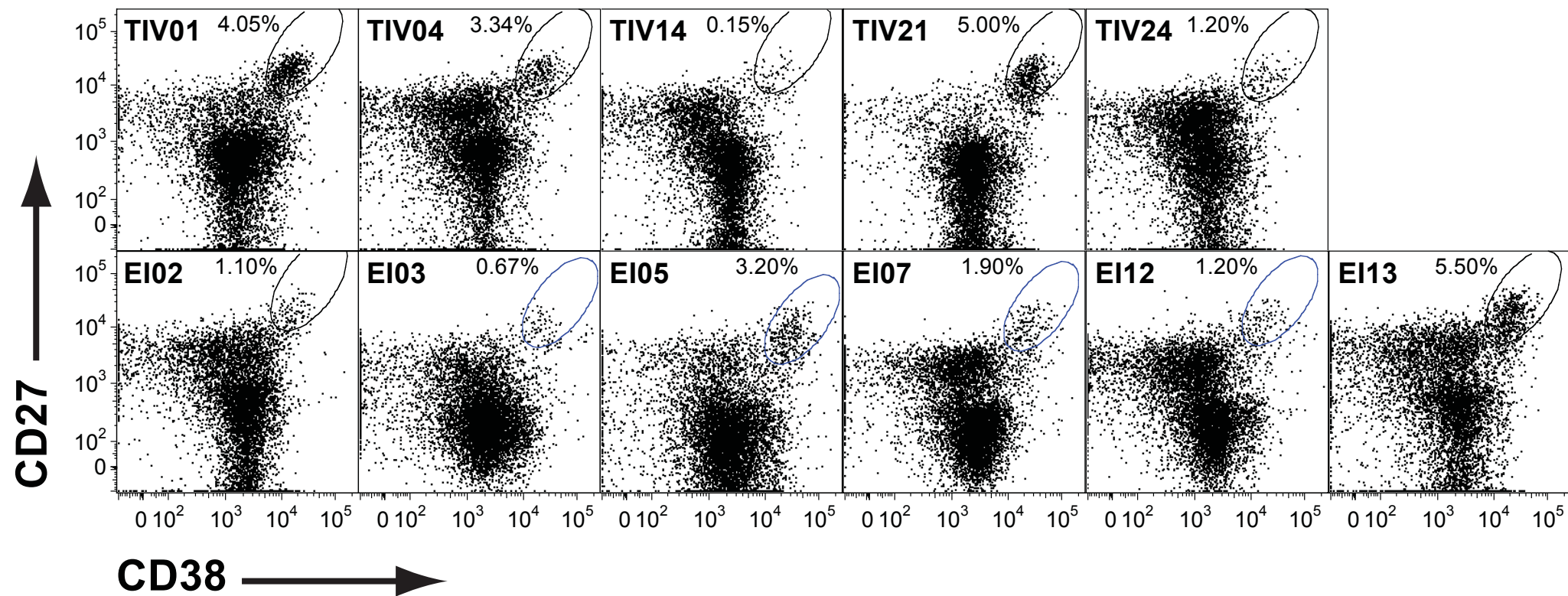

**Figure S2**

Supplement: Figure S2 — Plasmacytosis following TIV or EI. Plots shown are of total B cell populations (CD3/14/16/235a− CD19+) and are normalized to 10000 events per panel. Ellipse in upper right corner of each panel is homologous to the sorting gate used for the isolation of single plasma cells for mAb generation. Additional gating on CD20 was also performed for cell sorting and the final population sorted for mAb production was CD3/14/16/235a− CD19+ CD20−/lo CD27hi CD38hi. Percentages shown are of plasma cells as a fraction of total B cells. (PDF) [file pone.0025797.s003.pdf]

**A**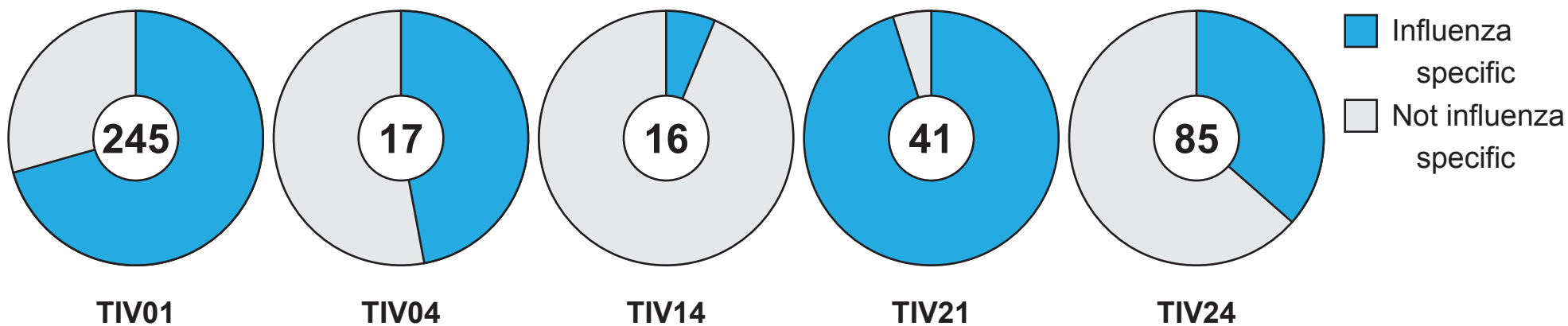**B**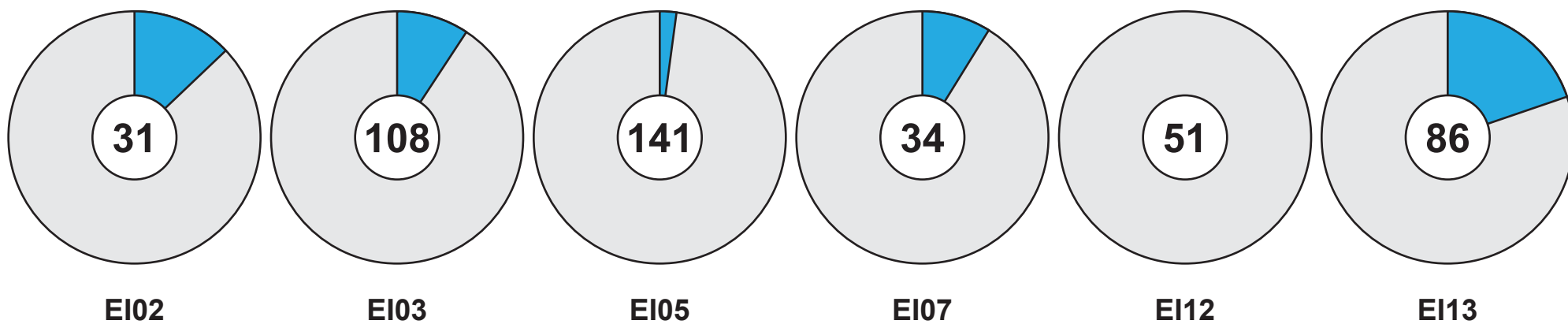**Figure S3**

Supplement: Figure S3 — Reactivities of human rmAbs recovered from TIV and EI. A. Antibodies from TIV subjects. We recovered plasma cells producing rmAbs against influenza antigens from all TIV subjects. In two subjects (TIV01 and TIV21) the majority of recovered rmAbs were reactive with influenza [179/245 (73%) and 39/41 (95%), respectively]. In the other three subjects, less than half of recovered rmAbs were influenza-specific [TIV04 8/17 (47%), TIV14 1/16 (6%), TIV24 35/85 (41%)]. B. Antibodies from EI subjects. We recovered plasma cells producing rmAbs against influenza antigens from five of six EI subjects; from one subject (EI12) we recovered 51 rmAbs that were not reactive for any antigen tested. None of the other EI subjects had more than 25% of rmAbs reactive with influenza [EI02 4/31 (13%), EI03 11/108 (10%), EI05 3/141 (2.1%), EI07 3/34 (9%), EI13 18/86 (21%)]. (PDF) [file pone.0025797.s004.pdf]

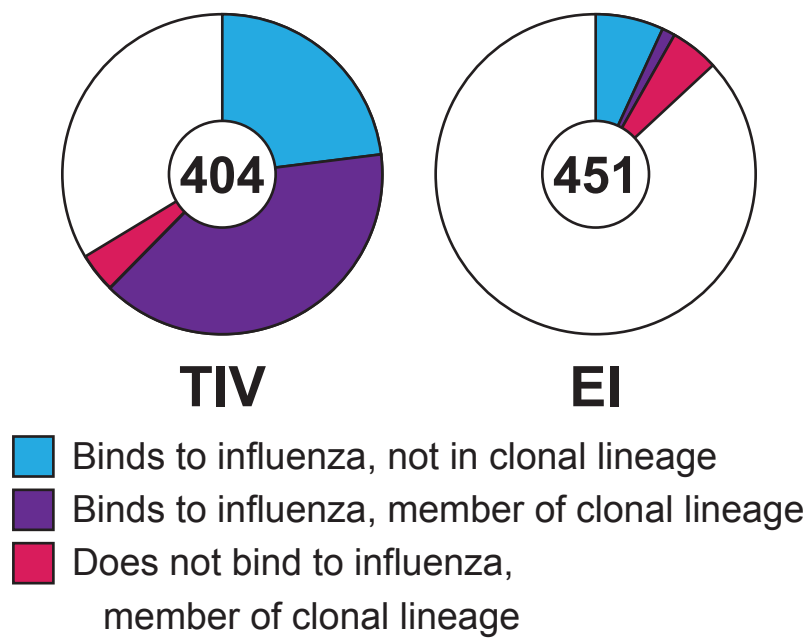

**Figure S4**

Supplement: Figure S4 — Relationship between influenza reactivity and clonal lineages from TIV and EI subjects. Influenza-specific rmAbs recovered from TIV were more likely to be in a clonal lineage compared with EI. In TIV, 93/404 (23%) of rmAbs were influenza-specific but not a member of a clonal lineage (blue wedge), 159/404 (39%) were both influenza-specific and members of clonal lineages (purple wedge), while only 16/404 (4%) were members of clonal lineages but not reactive with influenza antigens (red wedge). In EI, 31/451 (6.9%) were influenza-specific but not part of a clonal lineage, 6/451 (1.3%) were both influenza-specific and part of a clonal lineage, and 22/451 (4.9%) were not influenza specific but were part of a clonal lineage. (PDF) [file pone.0025797.s005.pdf]

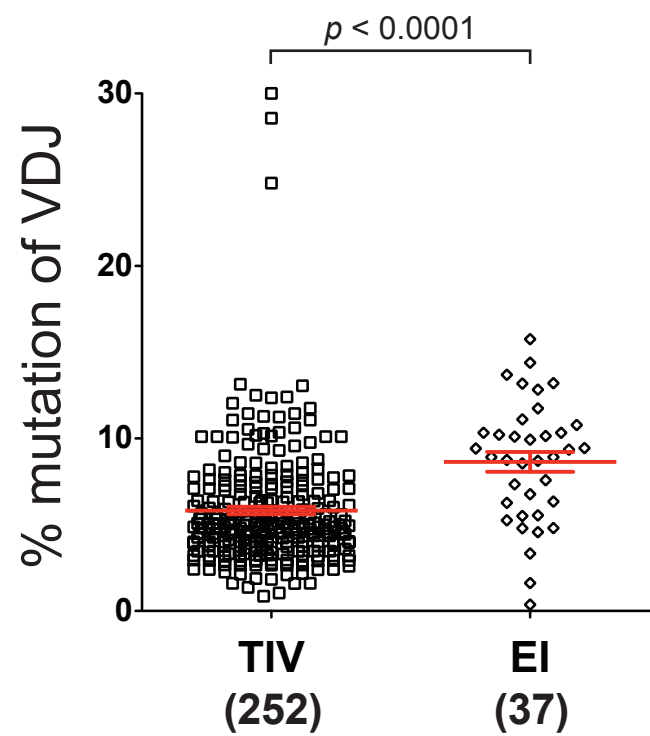

**Figure S5**

Supplement: Figure S5 — VDJ mutation rate of influenza-specific mAbs from TIV and EI subjects. VDJ mutation rates in rmAbs from TIV subjects (range 0.9–30.3%, mean 5.8%±0.2%) were lower on average than rmAbs from EI subjects (range 0.4–15.8%, mean 8.6%±0.6%) (two-tailed t-test, p<0.0001). (PDF) [file pone.0025797.s006.pdf]

# A: VDJ Mutation in TIV by Isotype

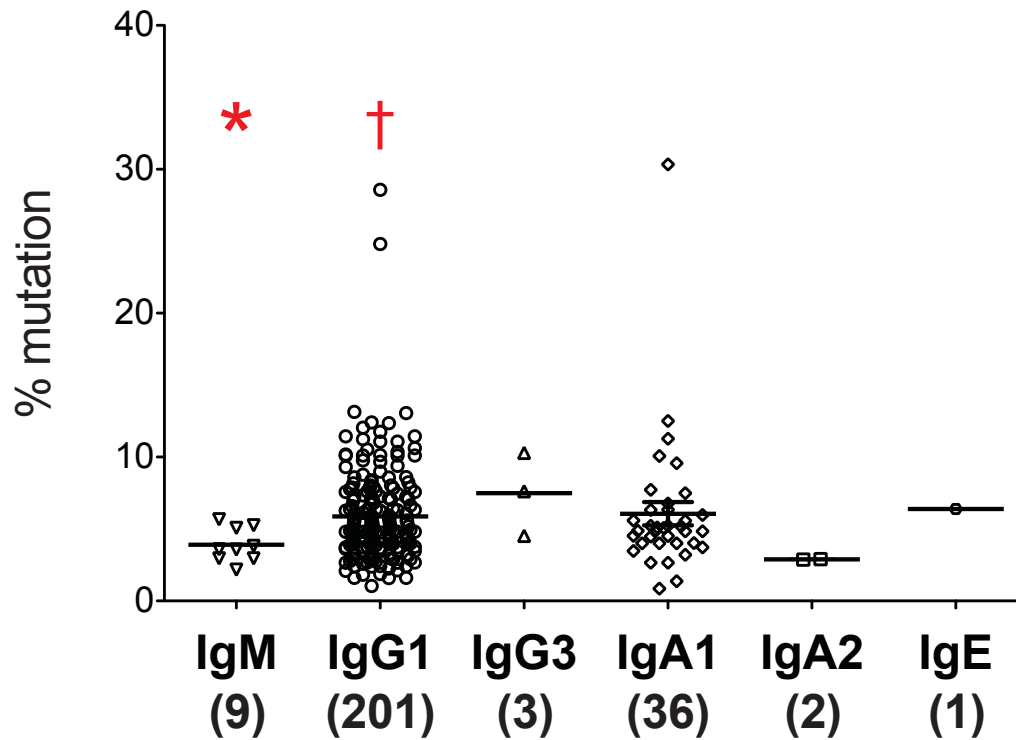

# B: VDJ Mutation in EI by Isotype

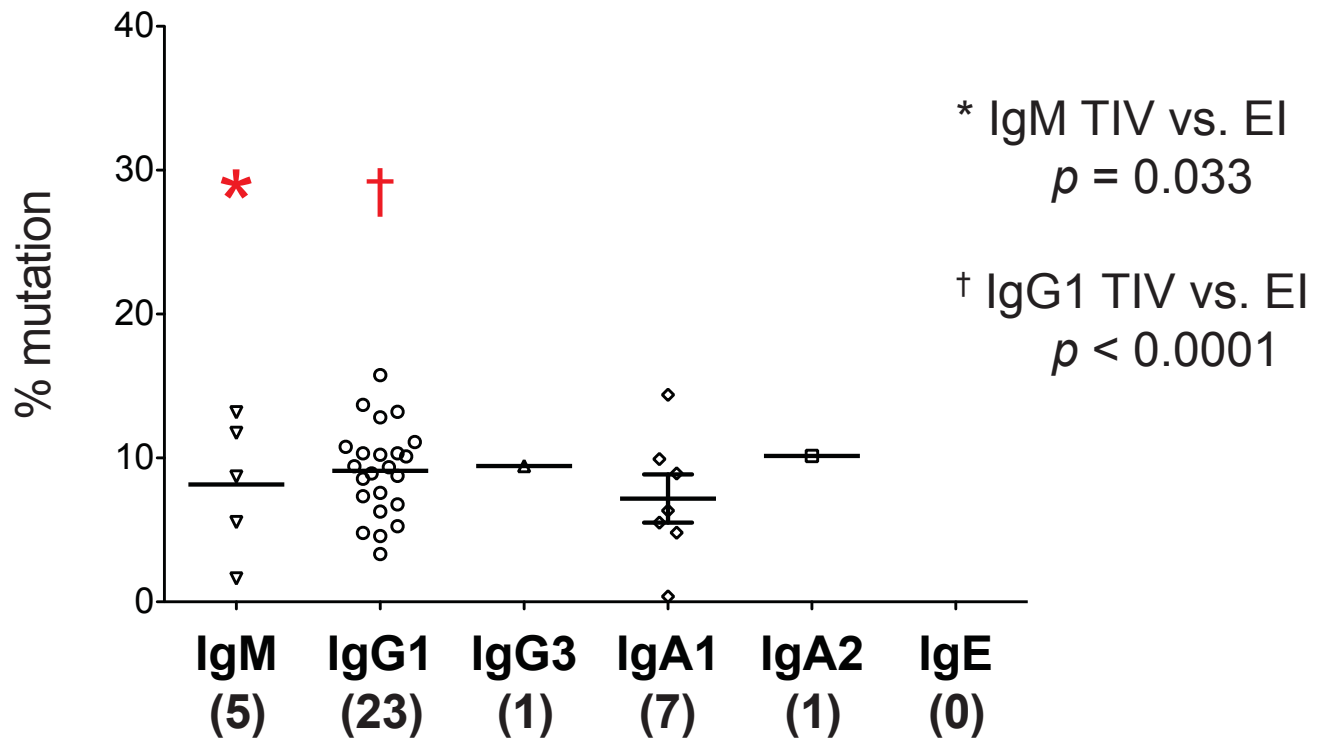

Figure S6

Supplement: Figure S6 — VDJ mutation rate of influenza-specific mAbs from TIV and EI subjects by isotype of mAb. A. HC isotype of influenza-specific rmAbs from TIV subjects were found to be predominantly IgG1 (201/252, 80%), followed by IgA1 (36/252, 14%) and IgM (9/252, 3.6%). VDJ mutation rates for these three isotypes were as follows: IgG1 5.9%±0.2%, IgA1 6.1%±0.8%, IgM 3.9%±0.4%. Comparison of mutation rates between isotypes within the TIV group did not show any significant differences. B. HC isotype of influenza-specific rmAbs from EI subjects were also found to be predominantly IgG1 (23/37, 62%), followed by IgA1 (7/37, 19%) and IgM (5/37, 14%). VDJ mutation rates for these three isotypes were as follows: IgG1 9.1%±0.7%, IgA1 7.2%±1.7%, IgM 8.2%±2.1%. Comparison of mutation rates between isotypes within the EI group did not show any significant differences. When compared between the TIV and EI groups, however, mutation rates for both IgG1 and IgM were found to be higher in rmAbs derived from EI subjects vs. those derived from TIV subjects (two-tailed t-test, p<0.0001 for IgG1, p = 0.033 for IgM). (PDF) [file pone.0025797.s007.pdf]

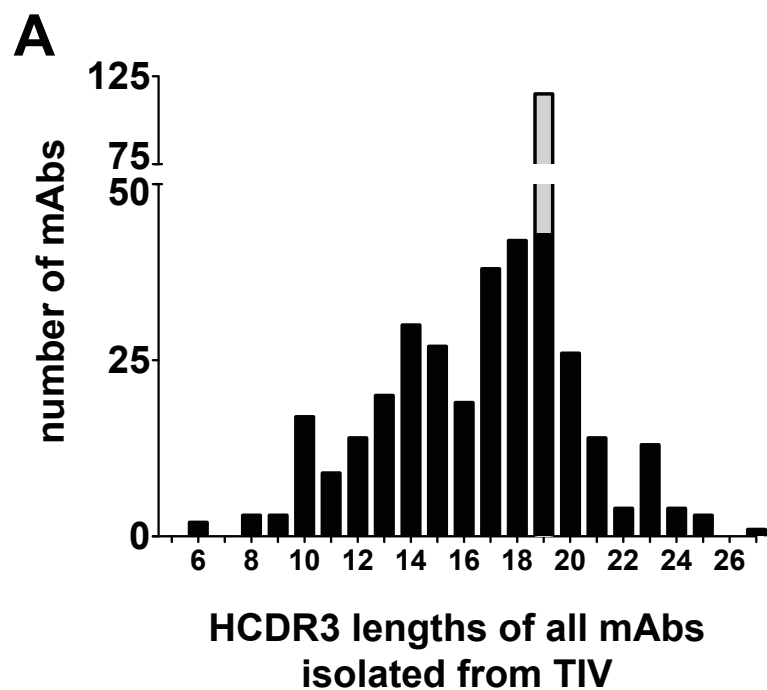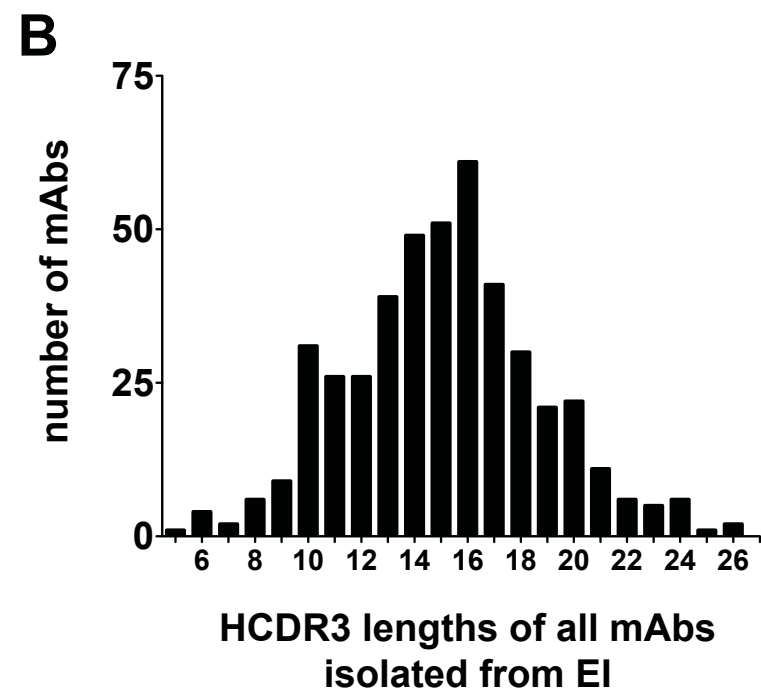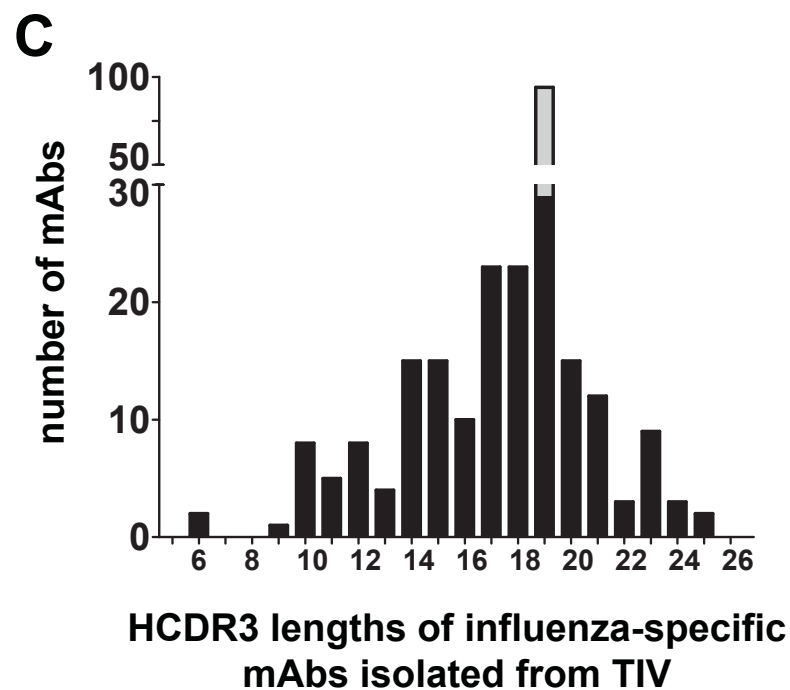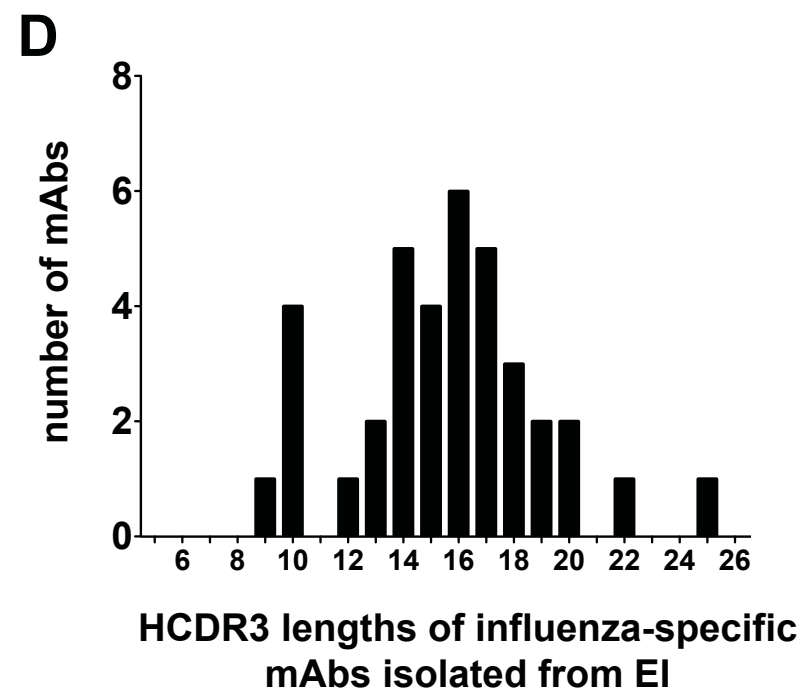

**Figure S7**

Supplement: Figure S7 — HC CDR3 length distribution of mAbs from TIV and EI subjects. A. HC CDR3 length distribution of all mAbs isolated from TIV subjects showed a predominance of mAbs with length 19; as with influenza-specific mAbs (Fig. S7C online) a large portion of this was contributed by 13 clonal lineages from one subject (TIV01) that had similar heavy chain rearrangements (VH4-59–JH6) but that did not share light chains. These clonal lineages contributed 68 mAbs (gray portion of bar). B. HC CDR3 length distribution of all mAbs isolated from EI subjects showed a distribution similar to that of the influenza-specific rmAbs (Fig. S7D online). As with influenza-specific mAbs, HC CDR3 lengths of 16 were the most common overall. Kolmogorov-Smirnov test of the distributions in A and B showed a difference in distribution (test statistic = 4.67, p<0.0001). C. HC CDR3 length distribution of influenza-specific rmAbs from TIV subjects. The number of aas in HC CDR3 was most commonly 19; 13 clonal lineages from subject TIV01 with similar rearrangements (VH4-59–JH6) contributed 65 rmAbs to this peak (gray portion of bar). D. HC CDR3 length distribution of influenza-specific rmAbs from EI subjects. HC CDR3 lengths of 16 were most common. Kolmogorov-Smirnov test of the distributions in C and D showed a difference in distribution (test statistic = 2.25, p<0.0001). (PDF) [file pone.0025797.s008.pdf]

TIV01 (173 mAbs)

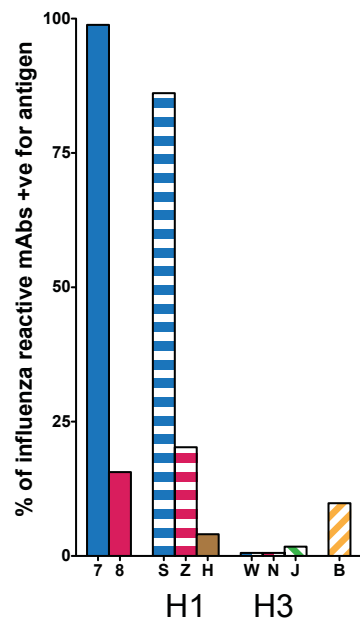

TIV04 (8 mAbs)

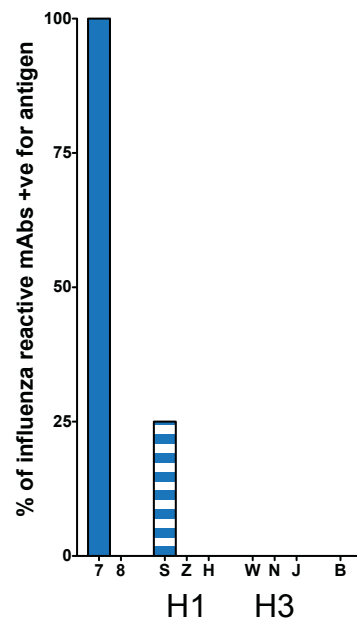

TIV21 (39 mAbs)

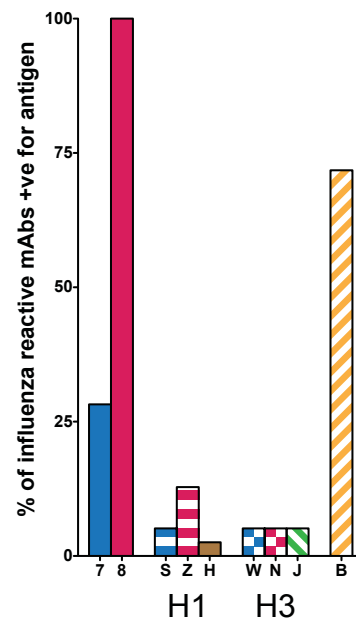

TIV24 (31 mAbs)

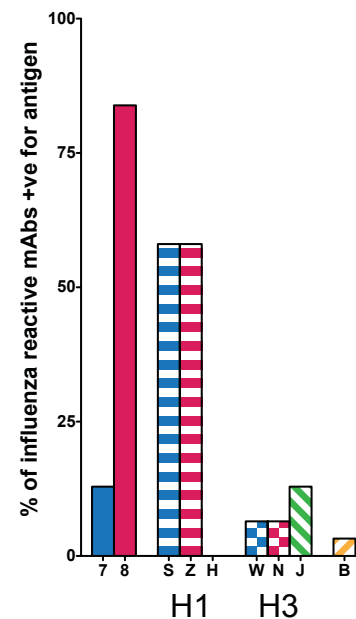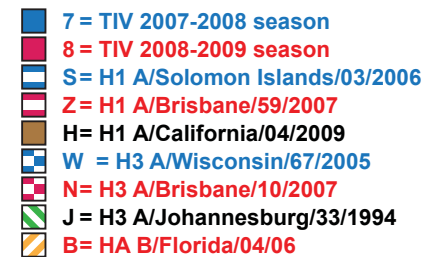

EI02 (4 mAbs)

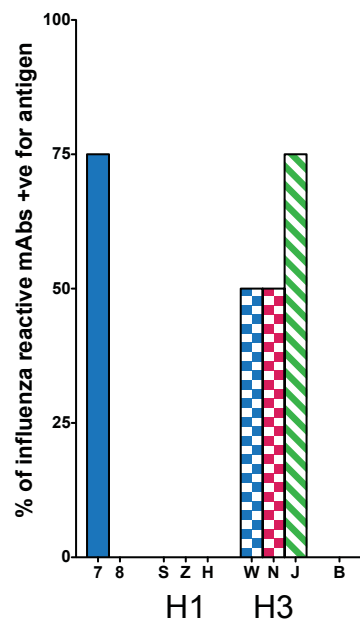

EI03 (10 mAbs)

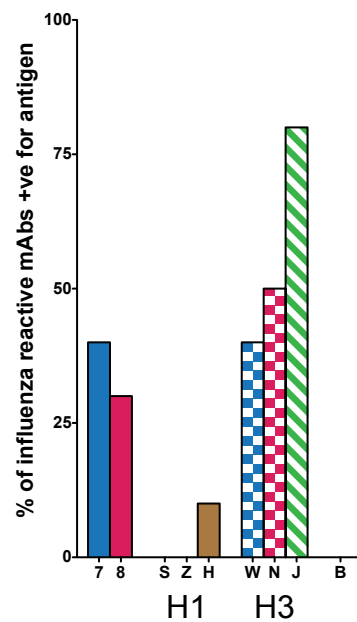

EI05 (3 mAbs)

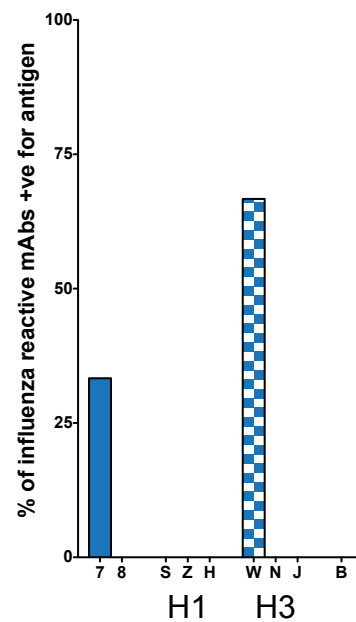

EI07 (3 mAbs)

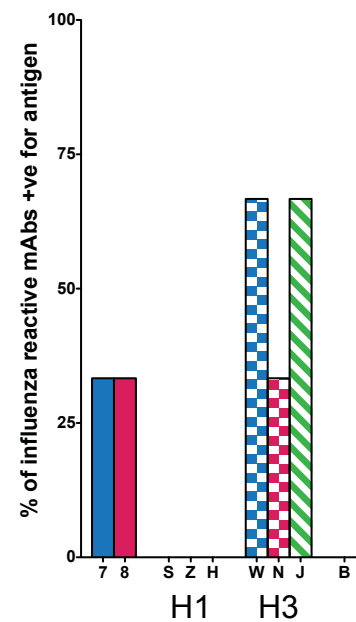

EI13 (17 mAbs)

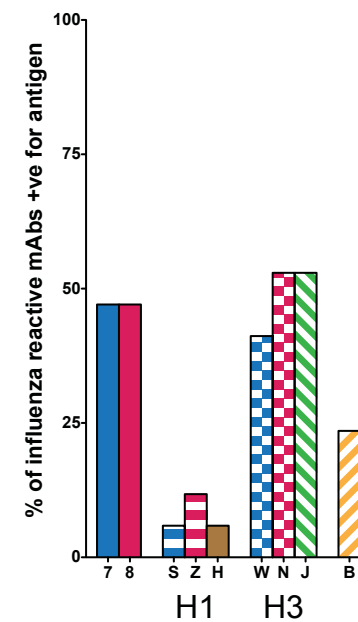

Figure S8

Supplement: Figure S8 — Distribution of rmAb reactivities among influenza-specific rmAbs from TIV and EI subjects. For each tested antigen, influenza-specific rmAbs were counted to determine how many rmAbs reacted with that antigen. In contrast to the other analyses, each reactivity was counted separately (e.g., rmAb reactive with 2007–2008 influenza vaccine and with H3 A/Wisconsin/67/2005 was counted as positive for both columns). Multiply reactive rmAbs were counted as positive for each antigen with which they reacted; bars do not sum to 100% for this analysis. For each TIV subject, rmAb reactivity was found to be primarily specific for individual strains. TIV01 (173 influenza-specific rmAbs): 171/173 (98.9%) reacted with 2007–2008 influenza vaccine, 149/173 (86%) reacted with H1 A/Solomon Islands/03/2006 while only 1/173 (0.6%) reacted with H3 A/Wisconsin/67/2005. SPR testing of rmAbs derived from subject TIV01 bound to rHA H1 A/Solomon Islands/03/2006 but not to H3 A/Wisconsin/67/2005 (Figs. S9A, S9B, S9C online). TIV04 (8 influenza-specific rmAbs): 8/8 (100%) reacted with 2007–2008 influenza vaccine, 2/8 (25%) reacted with H1 A/Solomon Islands/03/2006; no other reactivities detected. TIV14 omitted from this analysis as there was only one influenza-specific rmAb isolated (this rmAb reacted with 2007–2008 influenza vaccine, H1 A/Solomon Islands/03/2006, and H1 A/Brisbane/59/2007). TIV21 (39 influenza-specific rmAbs): 39/39 (100%) reacted with 2008–2009 influenza vaccine, 28/39 (72%) reacted with HA B/Florida/04/2006, while only 5/39 (13%) reacted with H1 A/Brisbane/59/2007 and 2/39 (5%) reacted with H3 A/Brisbane/10/2007. TIV24 (31 influenza-specific rmAbs): 26/31 (84%) reacted with 2008–2009 influenza vaccine, 18/31 (58%) reacted with H1 A/Brisbane/59/2007, while only 2/31 (6%) reacted with H3 A/Brisbane/10/2007 and 1/31 (3%) reacted with HA B/Florida/04/2006. Additionally, 18/31 (58%) reacted with H1 A/Solomon Islands/03/2006, however, only 4/31 (13%) reacted with 2007–2008 influenza [file pone.0025797.s009.pdf]

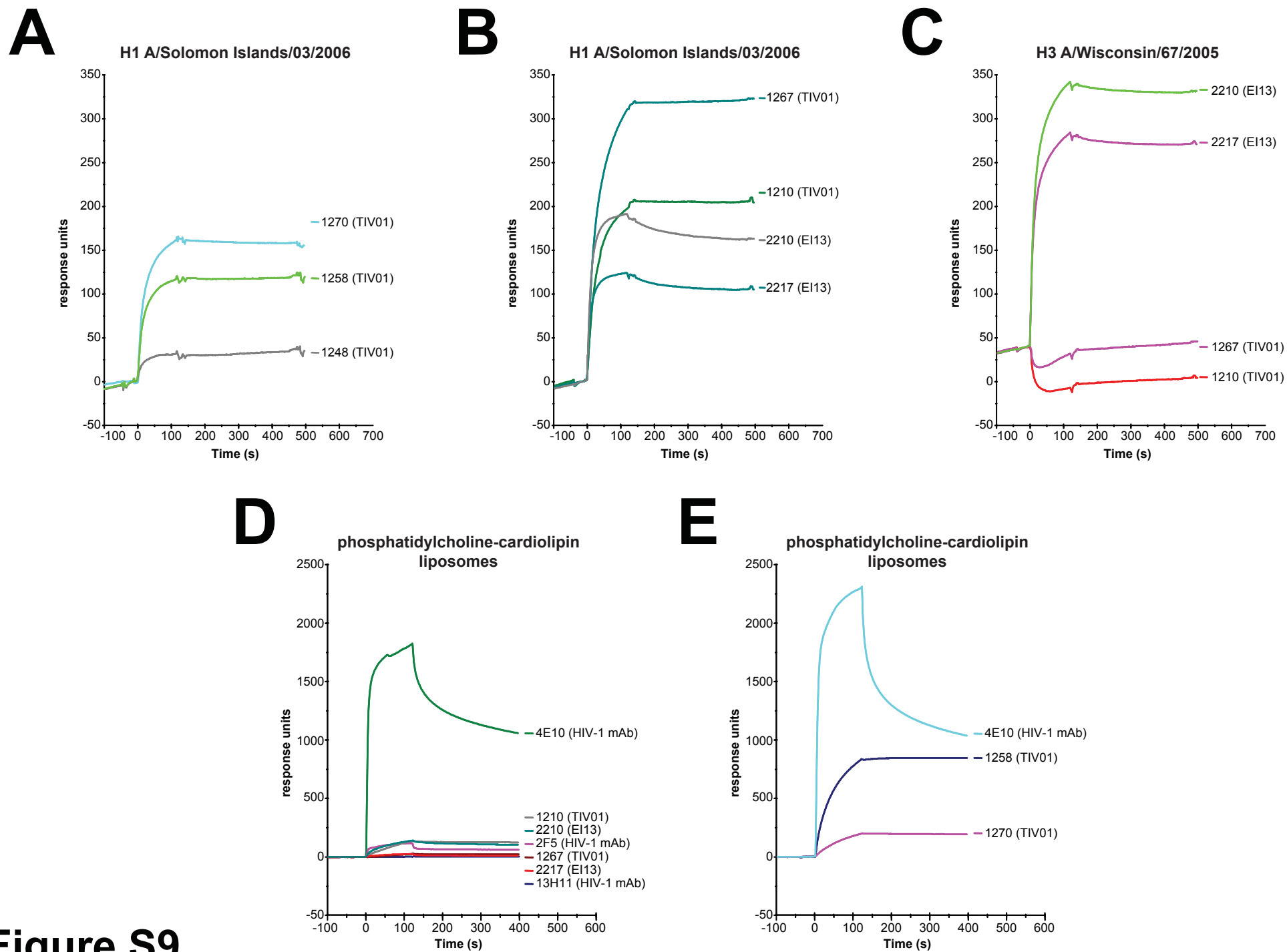

**Figure S9**

Supplement: Figure S9 — Surface plasmon resonance analysis of rmAbs recovered from TIV and EI subjects. A. Human rmAbs 1248, 1258, and 1270 from subject TIV01 bound to H1 A/Solomon Islands/03/2006 bound to an SPR chip. B. Human rmAbs 1210 and 1267 from subject TIV01 and rmAbs 2210 and 2217 from subject EI13 bound to H1 A/Solomon Islands/03/2006 bound to an SPR chip. C. Human rmAbs 1267 and 1210 from subject TIV01 did not bind to H3 A/Wisconsin/67/2005 bound to an SPR chip while rmAbs 2210 and 2217 from subject EI13 did bind. D. Human rmAbs from subjects TIV01 (1210 and 1267) and EI13 (2210 and 2217) showed essentially no interaction with phosphatidylcholine-cardiolipin liposomes. Anti-HIV-1 mAb 4E10 is shown as a positive control and anti-HIV-1 mAbs 2F5 and 13H11 are shown as negative controls. E. Human rmAb 1258 from subject TIV01 shows some degree of binding to phosphatidylcholine-cardiolipin liposomes while rmAb 1270 from subject TIV01 shows a lesser degree of binding. Anti-HIV-1 mAb 4E10 is shown as a positive control. No binding of rmAbs to apoferritin (control protein) or to phosphatidylcholine-phosphatidylserine liposomes was seen (data not shown). (PDF) [file pone.0025797.s010.pdf]
