# Supplementary material for: H3N2 Influenza Infection Elicits More Cross-Reactive and Less Clonally Expanded Anti-Hemagglutinin Antibodies Than Influenza Vaccination
Source: PLoS One. 2011 Oct 19;6(10):e25797. doi: 10.1371/journal.pone.0025797 (PMC3198447; doi:10.1371/journal.pone.0025797)
Supplement: Figure S10 — Additional representative clonal lineage of rmAbs from subject TIV01. Data for starred rmAbs appear in Table S12 online. Clonal lineage 643 from subject TIV01. Sixteen of 17 rmAbs (94%) bound one antigen, 1/17 (6%) was not influenza-specific. Four rmAbs were tested in additional assays. Affinity for rHA binding was measured for three rmAbs and all had sub-nanomolar affinity for rHA H1 A/Solomon Islands/03/2006 and no binding to H3 A/Wisconsin/67/2005. Neutralization assays for one rmAb (1267 IgG1) showed potent neutralization of H1N1 A/Solomon Islands/03/2006, weak neutralization of H1N1 A/Brisbane/59/2007 and no neutralization of H3N2 A/Wisconsin/67/2005. Two other rmAbs were tested only against H1N1 A/Brisbane/59/2007 and neither neutralized. All four tested rmAbs showed potent HAI against H1N1 A/Solomon Islands/03/2006; two rmAbs showed weak HAI activity against H3N2 A/Wisconsin/67/2005. (PDF) [file pone.0025797.s011.pdf]

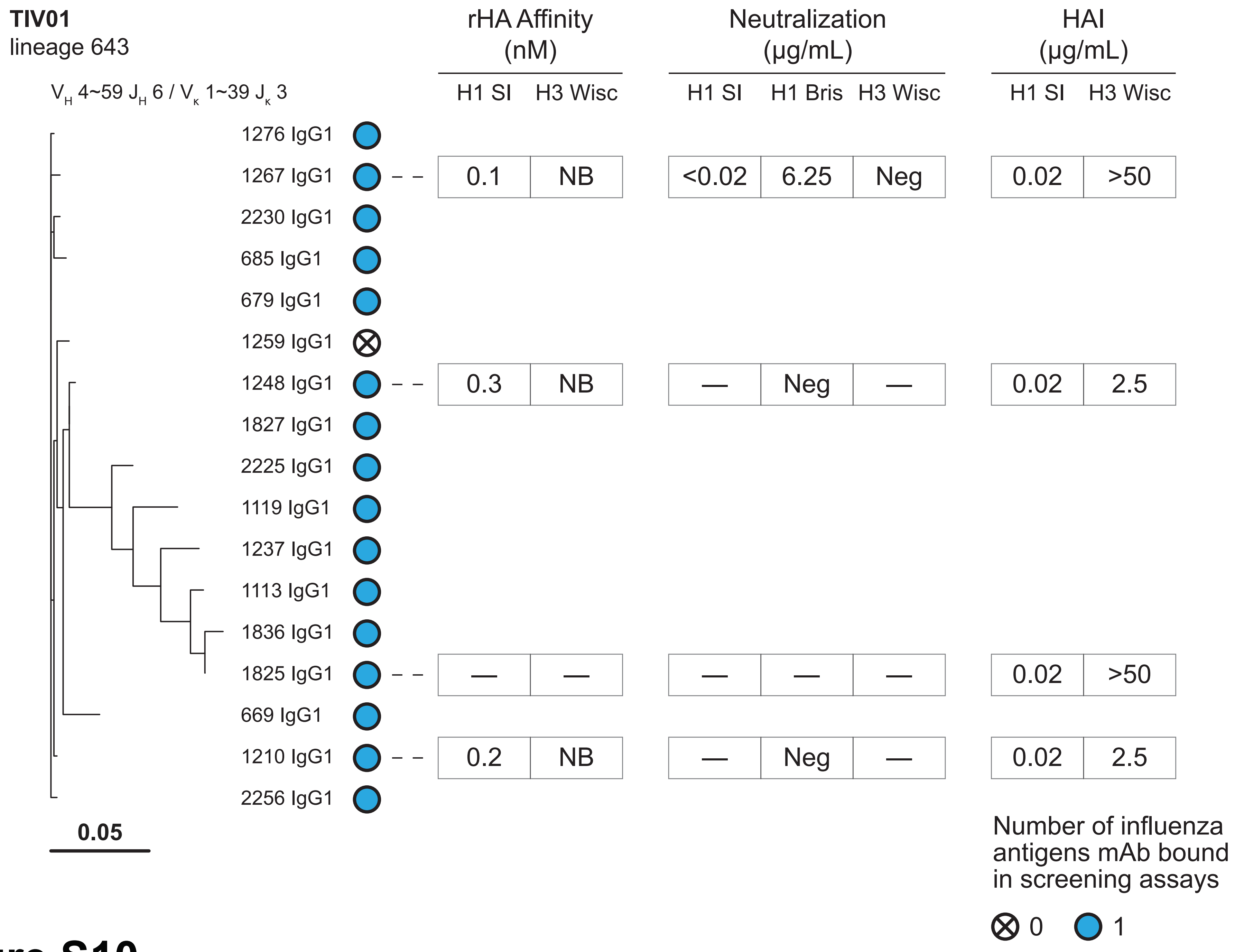
TIV01
lineage 643
V_H 4~59 J_H 6 / V_κ 1~39 J_κ 3
rHA Affinity (nM)
Neutralization (µg/mL)
HAI (µg/mL)
H1 SI
H3 Wisc
H1 SI
H1 Bris
H3 Wisc
H1 SI
H3 Wisc
1276 IgG1
1267 IgG1
0.1 NB <0.02 6.25 Neg 0.02 >50
2230 IgG1
685 IgG1
679 IgG1
1259 IgG1
1248 IgG1
0.3 NB — Neg — 0.02 2.5
1827 IgG1
2225 IgG1
1119 IgG1
1237 IgG1
1113 IgG1
1836 IgG1
1825 IgG1
— — — — — 0.02 >50
669 IgG1
1210 IgG1
0.2 NB — Neg — 0.02 2.5
2256 IgG1
0.05
Number of influenza antigens mAb bound in screening assays
0
1

Figure S10
