# Supplementary material for: H3N2 Influenza Infection Elicits More Cross-Reactive and Less Clonally Expanded Anti-Hemagglutinin Antibodies Than Influenza Vaccination
Source: PLoS One. 2011 Oct 19;6(10):e25797. doi: 10.1371/journal.pone.0025797 (PMC3198447; doi:10.1371/journal.pone.0025797)
Supplement: Figure S11 — Additional representative clonal lineages of rmAbs from TIV subjects. A. Clonal lineage 690 from subject TIV01. Three of 12 rmAbs (25%) bound one antigen, 8/12 (67%) bound two antigens, 1/12 (8%) bound none. B. Clonal lineage 2737 from subject TIV21. Five of 8 rmAbs (63%) bound one antigen, 3/8 (37%) bound two. (PDF) [file pone.0025797.s012.pdf]

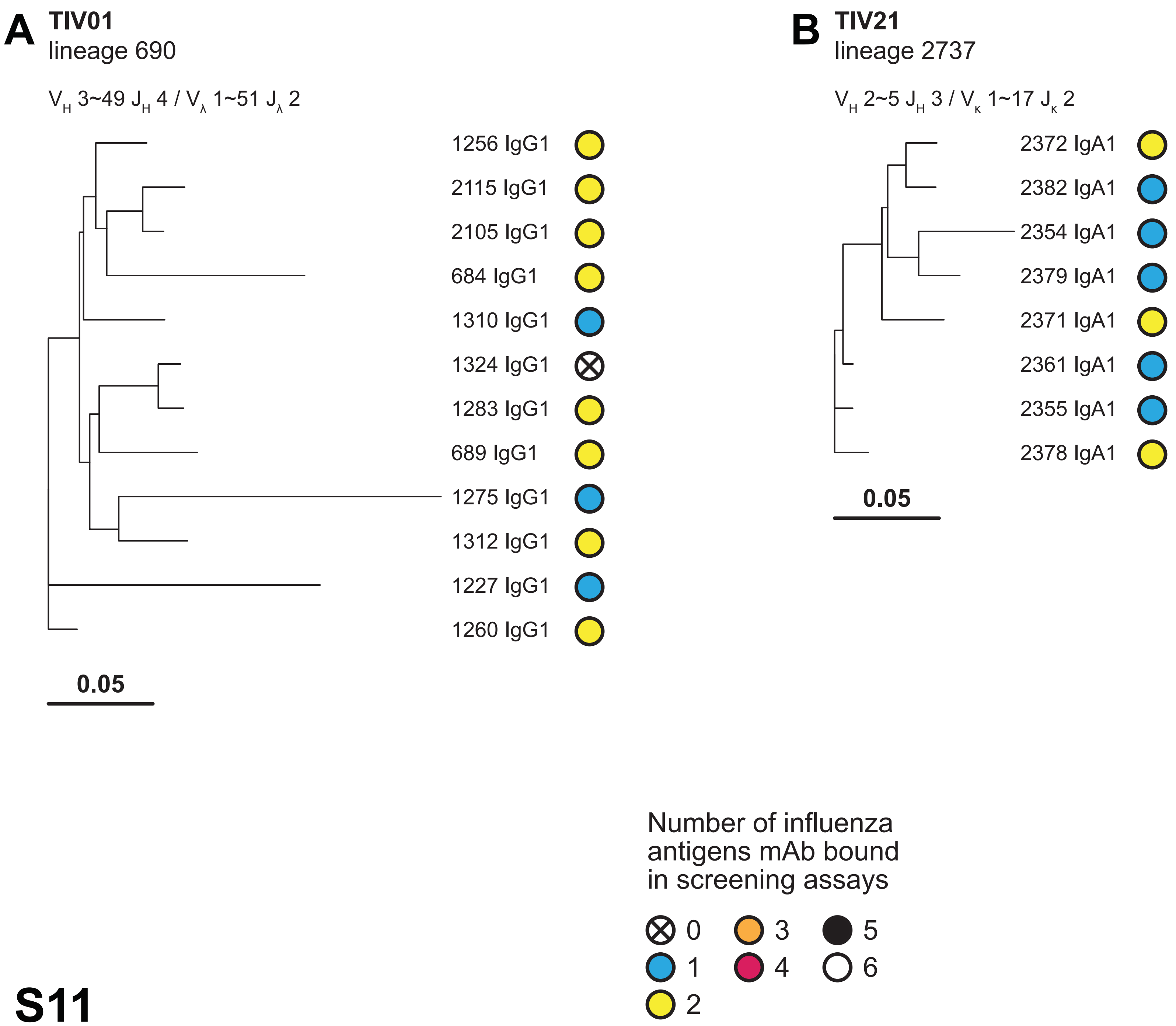

A
TIV01
lineage 690
V_H 3~49 J_H 4 / V_λ 1~51 J_λ 2
1256 IgG1
2115 IgG1
2105 IgG1
684 IgG1
1310 IgG1
1324 IgG1
1283 IgG1
689 IgG1
1275 IgG1
1312 IgG1
1227 IgG1
1260 IgG1
0.05
B
TIV21
lineage 2737
V_H 2~5 J_H 3 / V_κ 1~17 J_κ 2
2372 IgA1
2382 IgA1
2354 IgA1
2379 IgA1
2371 IgA1
2361 IgA1
2355 IgA1
2378 IgA1
0.05
Number of influenza antigens mAb bound in screening assays
0
1
2
3
4
5
6

Figure S11
