# Supplementary material for: H3N2 Influenza Infection Elicits More Cross-Reactive and Less Clonally Expanded Anti-Hemagglutinin Antibodies Than Influenza Vaccination
Source: PLoS One. 2011 Oct 19;6(10):e25797. doi: 10.1371/journal.pone.0025797 (PMC3198447; doi:10.1371/journal.pone.0025797)
Supplement: Figure S12 — Sequence alignment of H3 HAs used in this study. Amino acid sequences for H3 HA strains used in this study were downloaded from PubMed and aligned to the H3 A/Wisconsin/67/2005 sequence. Differences are highlighted in colors for each aligned sequence. The H3 A/Johannesburg/33/1994 strain was the least similar with 88.8% identity. Only the HA1 sequence for the Johannesburg strain was available; all sequences were aligned in this region only. (PDF) [file pone.0025797.s013.pdf]

Influenza A H3 Hemagglutinin Alignments

```
H3Wisc = H3 A/Wisconsin/67/2005     GenBank: ABO37569.1
H3Jobg = H3 A/Johannesburg/33/1994  GenBank: AAT64855.1
H3Cal  = H3 A/California/7/2004     GenBank: ABO37490.1
H3NY   = H3 A/New York/55/2004      GenBank: ACF41900.1
H3Bris = H3 A/Brisbane/10/2007      GenBank: ABW23353.1
H3Urgy = H3 A/Uruguay/716/2007      GenBank: ACD47215.1
H3Vict = H3 A/reassortant/IVR-155(Victoria/210/2009 x Puerto Rico/8/1934)
                                    GenBank: ADI52838.1
```

```
H3Wisc    QKLPGNDNST ATLCLGHHAV PNGTIVKTIT NDQIEVTNAT ELVQSSSTGG ICDSPHQILD    60
H3Jobg    QKLPGNDNST ATLCLGHHAV PNGTLVKTIT NDQIEVTNAT ELVQSSPTGR ICDSPHRILD    60
H3Cal     QKLPGNDNST ATLCLGHHAV PNGTIVKTIT NDQIEVTNAT ELVQSSSTGG ICDSPHQILD    60
H3NY      QKLPGNDNST ATLCLGHHAV PNGTIVKTIT NDQIEVTNAT ELVQSSSTGG ICDSPHQILD    60
H3Bris    QKLPGNDNST ATLCLGHHAV PNGTIVKTIT NDQIEVTNAT ELVQSSSTGE ICDSPHQILD    60
H3Urgy    QKLPGNDNST ATLCLGHHAV PNGTIVKTIT NDQIEVTNAT ELVQSSSTGE ICDSPHQILD    60
H3Vict    QKLPGNDNST ATLCLGHHAV PNGTIVKTIT NDQIEVTNAT ELVQNSSTGE ICDSPHQILD    60

H3Wisc    GENCTLIDAL LGDPQCDGFQ NKKWDLFVER SKAYSNCYPY DVPDYASLRS LVASSGTLEF   120
H3Jobg    GKNCTLIDAL LGDPHCDGFQ NKEWDLFVER SKAYSNCYPY DVPDYASLRS LVASSGTLEF   120
H3Cal     GENCTLIDAL LGDPQCDGFQ NKKWDLFVER SKAYSNCYPY DVPDYASLRS LVASSGTLEF   120
H3NY      GENCTLIDAL LGDPQCDGFQ NKKWDLFVER SKAYSNCYPY DVPDYASLRS LVASSGTLEF   120
H3Bris    GENCTLIDAL LGDPQCDGFQ NKKWDLFVER SKAYSNCYPY DVPDYASLRS LVASSGTLEF   120
H3Urgy    GENCTLIDAL LGDPQCDGFQ NKKWDLFVER SKAYSNCYPY DVPDYASLRS LVASSGTLEF   120
H3Vict    GKNCTLIDAL LGDPQCDGFQ NKKWDLFVER SKAYSNCYPY DVPDYASLRS LVASSGTLEF   120

H3Wisc    NDESFNWTGV TQNGTSSACK RRSNNSFFSR LNWLTHLKFK YPALNVTMPN NEKFDKLYIW   180
H3Jobg    INENFNWTGV AQDGKSYACK RGSVNSFFSR LNWLHKLEYK YPALNVTMPN NGKFDKLYIW   180
H3Cal     NNESFNWTGV TQNGTSSSCK RRSNNSFFSR LNWLTHLKFK YPALNVTMPN NEKFDKLYIW   180
H3NY      NNESFNWTGV TQNGTSSSCK RRSNNSFFSR LNWLTHLKFK YPALNVTMPN NEKFDKLYIW   180
H3Bris    NNESFNWTGV TQNGTSSACI RRSNNSFFSR LNWLTHLKFK YPALNVTMPN NEKFDKLYIW   180
H3Urgy    NNESFNWTGV TQNGTSSSCI RRSNNSFFSR LNWLTHLKFK YPALNVTMPN NEKFDKLYIW   180
H3Vict    NNESFNWTGV TQNGTSSACI RRSKNSFFSR LNWLTNLNFK YPALNVTMPN NEQFDKLYIW   180

H3Wisc    GVHHPGTDND QIFLHAQASG RITVSTKRSQ QTVIPNIGSR PRIRNIPSRI SIYWTIVKPG   240
H3Jobg    GVHHPSTDSD QTSLYVRASG RVTVSTKRSQ QTVIPDIGYR PWVRGQSSRI SIYWTIVKPG   240
H3Cal     GVHHPGTNND QISLYTQASG RITVSTKRSQ QTVIPNIGSR PRVRDIPSRI SIYWTIVKPG   240
H3NY      GVHHPVTDND QIRLYAQASG RITVSTKRSQ QTVIPNIGSR PRVRDIPSRI SIYWTIVKPG   240
H3Bris    GVHHPGTDND QIFPYAQASG RITVSTKRSQ QTVIPNIGSR PRVRNIPSRI SIYWTIVKPG   240
H3Urgy    GVHHPGTDND QIFLYAQASG RITVSTKRSQ QTVIPNIGSR PRVRNIPSRI SIYWTIVKPG   240
H3Vict    GVHHPVTDKD QIFLYAQASG RITVSTKRSQ QTVIPNIGSR PRVRNIPSRI SIYWTIVKPG   240

H3Wisc    DILLINSTGN LIAPRGYFKI RSGKSSIMRS DAPIGKCNSE CITPNGSIPN DKPFQNVNRI   300
H3Jobg    DILLINSTGN LIAPRGYFKI RNGKSSIMRS DAPIGNCSSE CITPNGSIPN DKPFQNVNRI   300
H3Cal     DILLINSTGN LIAPRGYFKI RSGKSSIMRS DAPIGKCNSE CITPNGSIPN DKPFQNVNRI   300
H3NY      DILLINSTGN LIAPRGYFKI RSGKSSIMRS DAPIGKCNSE CITPNGSIPN DKPFQNVNRI   300
H3Bris    DILLINSTGN LIAPRGYFKI RSGKSSIMRS DAPIGKCNSE CITPNGSIPN DKPFQNVNRI   300
H3Urgy    DILLINSTGN LIAPRGYFKI RSGKSSIMRS DAPIGKCNSE CITPNGSIPN DKPFQNVNRI   300
H3Vict    DILLINSTGN LIAPRGYFKM QSGKSSIMRS DAPIGKCNSE CITPNGSIPN DKPFQNVNRI   300

H3Wisc    TYGACPRYVK QNTLKLATGM RNVPEKQTRG IFGAIAGFIE NGWEGMVDGW YGFRH        355
H3Jobg    TYGACPRYVK QNTLKLATGM RNVPEKQTRG IFGAIAGFIE NGWEGMV                 347
H3Cal     TYGACPRYVK QNTLKLATGM RNVPEKQTRG IFGAIAGFIE NGWEGMVDGW YGFRH        355
H3NY      TYGACPRYVK QNTLKLATGM RNVPEKQTRG IFGAIAGFIE NGWEGMVDGW YGFRH        355
H3Bris    TYGACPRYVK QNTLKLATGM RNVPEKQTRG IFGAIAGFIE NGWEGMVDGW YGFRH        355
H3Urgy    TYGACPRYVK QNTLKLATGM RNVPEKQTRG IFGAIAGFIE NGWEGMVDGW YGFRH        355
H3Vict    TYGACPRYVK QNTLKLATGM RNVPEKQTRG IFGAIAGFIE NGWEGMVDGW YGFRH        355
```

Homology with H3Wisc

```
H3Jobg    308 / 347 = 88.8%
H3Cal     347 / 355 = 97.7%
H3NY      348 / 355 = 98.0%
H3Bris    349 / 355 = 98.3%
H3Urgy    349 / 355 = 98.3%
H3Vict    340 / 355 = 95.8%
```


Figure S12
