# Supplementary material for: H3N2 Influenza Infection Elicits More Cross-Reactive and Less Clonally Expanded Anti-Hemagglutinin Antibodies Than Influenza Vaccination
Source: PLoS One. 2011 Oct 19;6(10):e25797. doi: 10.1371/journal.pone.0025797 (PMC3198447; doi:10.1371/journal.pone.0025797)
Supplement: Table S1 — Clonal lineages of antibodies from TIV subjects. (PDF) [file pone.0025797.s014.pdf]

Table S1. Clonal lineages of antibodies from TIV subjects.

Lineage ID	Subject ID	Isotype	V_H	J_H	CDR H3 length	Light chain	V_L	J_L	CDR L3 length	Number of lineage members	Percent influenza reactive
860†	TIV01	G1	1-2	6	19	λ	3-21	2	11	3	100%
2461	TIV01	G1	3-7	5	17	λ	3-1	2	9	2	100%
1261	TIV01	G1	3-9	6	20	κ	1-33	4	9	4	75%
1250	TIV01	G1	3-15	3	12	λ	1-40	2	11	2	100%
1945	TIV01	G1	3-23	3	16	κ	3-15	4	10	2	100%
2155	TIV01	G1	3-30	3	15	λ	1-40	3	11	2	0%
693	TIV01	A1	3-30	6	17	κ	3-20	2	10	3	100%
688	TIV01	G1	3-30	6	17	λ	1-44	3	11	8	88%
648	TIV01	G1	3-30	6	24	κ	1-33	4	9	2	100%
1347	TIV01	G1	3-48	5	11	λ	3-21	1	11	2	100%
690*	TIV01	G1	3-49	4	18	λ	1-51	2	12	12	92%
1285	TIV01	G1	3-49	4	18	κ	1-33	4	9	2	0%
652	TIV01	G1	4-4	6	23	λ	3-19	2	12	9	88%
1329	TIV01	A1 (2), A2 (1)	4-30	6	18	κ	3-20	2	10	3	100%
1258	TIV01	G1	4-30	6	18	κ	3-20	2	10	2	50%
1255	TIV01	G1	4-30	6	18	κ	3-20	3	10	2	50%
1270	TIV01	G1 (1), A1 (1)	4-59	5	15	λ	2-14	1	10	2	100%
1310	TIV01	G1	4-59	6	20	λ	1-44	2	11	2	50%
1251	TIV01	G1 (1), A1 (1)	4-59	6	19	λ	1-44	3	11	2	100%
641*	TIV01	G1 (10), A1 (9)	4-59	6	19	κ	1-39	2	9	19	89%
649	TIV01	G1	4-59	6	19	κ	1-39	4	9	2	100%
1277	TIV01	G1 (3), A1 (1)	4-59	6	19	κ	3-20	1	8	4	100%
638	TIV01	G1	4-59	6	19	κ	1-39	4	12	2	100%
640	TIV01	G1	4-59	6	19	κ	1-17	1	9	3	100%
1342	TIV01	G1	4-59	6	19	κ	1-39	4	10	3	100%
1953	TIV01	G1	4-59	6	19	λ	3-21	2	12	2	100%
643*	TIV01	G1	4-59	6	19	κ	1-39	3	10	17	94%
639	TIV01	G1	4-59	6	19	κ	1-39	1	9	5	100%
687	TIV01	G1	4-59	6	19	κ	3-11	4	11	5	100%
1301	TIV01	G1	4-59	6	19	κ	1-39	2	9	2	100%
1332	TIV01	G1	4-59	6	19	κ	1-39	2	11	2	100%
1300	TIV01	G1 (2), A1 (1)	4-b	6	19	λ	7-43	3	11	2	50%
2775	TIV04	M	3-7	4	14	λ	1-44	3	11	4	100%
2774	TIV04	G1	4-31	4	20	λ	3-10	3	11	2	50%
2735	TIV21	G1	1-2	4	12	λ	3-21	2	10	2	100%
2731	TIV21	G1 (1), A1 (1)	1-2	5	14	κ	1-39	5	9	2	100%
2737*	TIV21	A1	2-5	3	19	κ	1-17	2	9	8	100%
2747	TIV21	G1	3-30	2	15	κ	1-39	1	9	2	100%
2740	TIV21	G1	4-39	1	15	κ	2-28	5	9	3	100%
2756	TIV21	G1	4-39	3	17	κ	1-5	1	9	3	100%
2940	TIV24	G1 (1), E (1)	3-7	1	10	λ	1-44	2	12	2	100%
2902	TIV24	G1	3-7	4	10	λ	1-44	2	12	5	100%
2897	TIV24	G1	3-13	6	21	κ	3-20	4	10	4	100%
2976	TIV24	G1	3-13	6	21	κ	3-20	4	10	2	100%
2899	TIV24	G1 (2), A1 (1)	3-23	3	15	κ	1-33	5	9	3	100%

No clonal lineages were identified in mAbs derived from subject TIV14.

*Trees for these clonal lineages appear in Figure 3 (lineage 641), Figure S9 online (lineage 643), and Figure S10 online (lineages 690 and 2737).

†Tree for this clonal lineage appears in Whittle J, Zhang R, Khurana S, King LR, Manischewitz J, Golding H, Dormitzer PR, Haynes BF, Walter EB, Moody MA, Kepler TB, Liao H-X, Harrison SC. Broadly neutralizing human antibody that recognizes the receptor-binding pocket of influenza virus hemagglutinin. *Proc Nat Acad Sci USA* in press (2011).

Antibodies from a given TIV subject were identified as being a clonal lineage if they met the following criteria.
1) Inferred heavy and light chain rearrangements used the same V and J segments.
2) CDR 3 length are identical for both heavy and light chains.
3) 70% or greater nucleotide identity within CDR 3 for both heavy and light chains.
