# Supplementary material for: H3N2 Influenza Infection Elicits More Cross-Reactive and Less Clonally Expanded Anti-Hemagglutinin Antibodies Than Influenza Vaccination
Source: PLoS One. 2011 Oct 19;6(10):e25797. doi: 10.1371/journal.pone.0025797 (PMC3198447; doi:10.1371/journal.pone.0025797)
Supplement: Table S2 — Clonal lineages of antibodies from EI subjects. (PDF) [file pone.0025797.s015.pdf]

Table S2. Clonal lineages of antibodies from EI subjects.

Lineage ID	Subject ID	Isotype	V_H	J_H	CDR H3 length	Light chain	V_L	J_L	CDR L3 length	Number of lineage members	Percent influenza reactive
2342	EI03	A1	3-9	6	21	λ	2-23	2	12	2	0%
1822	EI05	G3 (1), A2 (1)	3-23	4	10	κ	3-15	1	10	2	0%
1579	EI05	G3	3-23	5	15	κ	3-20	1	9	2	0%
1799	EI05	G3	3-74	4	10	λ	3-23	3	10	2	0%
1881	EI05	G3	4-61	3	16	λ	1-40	2	11	2	0%
1813	EI05	G1 (2), G3 (1)	4-61	3	16	λ	1-40	3	11	3	0%
1849	EI05	A1 (2), A2 (1)	4-61	4	14	λ	3-1	2	9	3	0%
1803	EI05	G1	5-51	3	20	κ	3-15	2	9	2	0%
1964	EI12	A2	3-48	4	7	λ	5-45	3	9	2	0%
2569*	EI13	M (3), A1 (1)	1-46	3	10	κ	2-24	2	9	4	100%
2566	EI13	G3 (1), A1 (1)	3-15	4	16	λ	1-47	3	10	2	0%
2582	EI13	G1	3-43	1	17	κ	1-5	1	9	2	100%

No clonal lineages were identified in mAbs derived from subjects EI02 and EI07.

*Tree for this clonal lineage appears in Figure 4.

Antibodies from a given TIV subject were identified as being a clonal lineage if they met the following criteria.
1) Inferred heavy and light chain rearrangements used the same V and J segments.
2) CDR 3 length are identical for both heavy and light chains.
3) 70% or greater nucleotide identity within CDR 3 for both heavy and light chains.
