# Supplementary material for: H3N2 Influenza Infection Elicits More Cross-Reactive and Less Clonally Expanded Anti-Hemagglutinin Antibodies Than Influenza Vaccination
Source: PLoS One. 2011 Oct 19;6(10):e25797. doi: 10.1371/journal.pone.0025797 (PMC3198447; doi:10.1371/journal.pone.0025797)
Supplement: Table S3 — Isotypes of isolated influenza-specific rmAbs. (PDF) [file pone.0025797.s016.pdf]

Table S3. Isotypes of isolated influenza-specific rmAbs.

Subject	Influenza-Specific rmAbs									
	Isotype									Total
	IgM	IgD	IgG1	IgG2	IgG3	IgG4	IgA1	IgA2	IgE	
	N (%)									N
TIV01	2 (1.1%)	-*	149 (85.6%)	-	2 (1.1%)	-	20 (11.5%)	1 (0.6%)	-	174
TIV04	5 (62.5%)	-	2 (25%)	-	-	-	-	1 (12.5%)	-	8
TIV14	-	-	1 (100%)	-	-	-	-	-	-	1
TIV21	1 (2.6%)	-	23 (59%)	-	1 (2.6%)	-	14 (35.9%)	-	-	39
TIV24	1 (3.2%)	-	27 (87.1%)	-	-	-	2 (6.5%)	-	1 (3.2%)	31
**total**	**9 (3.6%)**	**-**	**202 (79.8%)**	**-**	**3 (1.2%)**	**-**	**36 (14.2%)**	**2 (0.8%)**	**1 (0.4%)**	**253**
EI02	-	-	3 (75%)	-	-	-	1 (25%)	-	-	4
EI03	-	-	7 (70%)	-	-	-	3 (30%)	-	-	10
EI05	2 (66.7%)	-	1 (33.3%)	-	-	-	-	-	-	3
EI07	-	-	3 (100%)	-	-	-	-	-	-	3
EI12	-	-	-	-	-	-	-	-	-	-
EI13	3 (17.6%)	-	9 (52.9%)	-	1 (5.9%)	-	3 (17.6%)	1 (5.9%)	-	17
**total**	**5 (13.5%)**	**-**	**23 (62.2%)**	**-**	**1 (2.7%)**	**-**	**7 (18.9%)**	**1 (2.7%)**	**-**	**37**

* - = No antibodies of this isotype isolated.
