# Supplementary material for: H3N2 Influenza Infection Elicits More Cross-Reactive and Less Clonally Expanded Anti-Hemagglutinin Antibodies Than Influenza Vaccination
Source: PLoS One. 2011 Oct 19;6(10):e25797. doi: 10.1371/journal.pone.0025797 (PMC3198447; doi:10.1371/journal.pone.0025797)
Supplement: Table S4 — Isotypes of isolated rmAbs not specific for influenza antigens. (PDF) [file pone.0025797.s017.pdf]

Table S4. Isotypes of isolated rmAbs not specific for influenza antigens.

Subject	rmAbs Not Specific for Influenza									
	Isotype									
	IgM	IgD	IgG1	IgG2	IgG3	IgG4	IgA1	IgA2	IgE	Total
	N (%)									N
TIV01	9 (12.5%)	-*	47 (65.3%)	-	3 (4.2%)	-	13 (18.1%)	-	-	72
TIV04	3 (33.3%)	-	3 (33.3%)	-	-	-	2 (22.2%)	1 (11.1%)	-	9
TIV14	2 (13.3%)	-	3 (20%)	-	2 (13.3%)	-	8 (53.3%)	-	-	15
TIV21	1 (50%)	-	-	-	-	-	1 (50%)	-	-	2
TIV24	8 (14.8%)	-	17 (31.5%)	1 (1.9%)	6 (11.1%)	-	22 (40.7%)	-	-	54
**total**	**23 (15.1%)**	**-**	**70 (46.1%)**	**1 (0.7%)**	**11 (7.2%)**	**-**	**46 (30.3%)**	**1 (0.7%)**	**-**	**152**
EI02	11 (40.7%)	-	3 (11.1%)	-	2 (7.4%)	1 (3.7%)	10 (37.0%)	-	-	27
EI03	24 (24.5%)	-	22 (22.4%)	-	13 (13.3%)	-	37 (37.8%)	2 (2%)	-	98
EI05	19 (13.8%)	-	27 (19.6%)	-	44 (31.9%)	1 (0.7%)	40 (29%)	7 (5.1%)	-	138
EI07	6 (19.4%)	-	15 (48.4%)	-	2 (6.5%)	-	7 (22.6%)	1 (3.2%)	-	31
EI12	13 (25.5%)	-	13 (25.5%)	-	3 (5.9%)	-	12 (23.5%)	10 (19.6%)	-	51
EI13	19 (27.5%)	1 (1.4%)	10 (14.5-%)	-	8 (11.6%)	-	23 (33.3%)	8 (11.6%)	-	69
**total**	**92 (22.2%)**	**1 (0.2%)**	**90 (21.7%)**	**-**	**72 (17.4%)**	**2 (0.5%)**	**129 (31.2%)**	**28 (6.8%)**	**-**	**414**

* - = No antibodies of this isotype isolated.
