# Supplementary material for: H3N2 Influenza Infection Elicits More Cross-Reactive and Less Clonally Expanded Anti-Hemagglutinin Antibodies Than Influenza Vaccination
Source: PLoS One. 2011 Oct 19;6(10):e25797. doi: 10.1371/journal.pone.0025797 (PMC3198447; doi:10.1371/journal.pone.0025797)
Supplement: Table S5 — Heavy chain family usage of influenza-specific rmAbs. (PDF) [file pone.0025797.s018.pdf]

Table S5. Heavy chain family usage of influenza-specific rmAbs.

Subject	Influenza-Specific rmAbs							
	Heavy Chain Family							
	1	2	3	4	5	6	7	Total
	N (%)							N
TIV01	5 (2.9%)	-*	56 (32.2%)	113 (64.9%)	-	-	-	174
TIV04	-	-	6 (75%)	2 (25%)	-	-	-	8
TIV14	-	-	1 (100%)	-	-	-	-	1
TIV21	6 (15.4%)	8 (20.5%)	17 (43.6%)	8 (20.5%)	-	-	-	39
TIV24	2 (6.5%)	-	28 (90.3%)	1 (3.2%)	-	-	-	31
**total**	**13 (5.1%)**	**8 (3.2%)**	**108 (42.7%)**	**124 (49.0%)**	**-**	**-**	**-**	**253**
EI02	-	-	2 (50%)	2 (50%)	-	-	-	4
EI03	4 (40%)	2 (20%)	1 (10%)	2 (20%)	1 (10%)	-	-	10
EI05	1 (33.3%)	1 (33.3%)	-	1 (33.3%)	-	-	-	3
EI07	2 (66.7%)	-	1 (33.3%)	-	-	-	-	3
EI12	-	-	-	-	-	-	-	0
EI13	7 (41.2%)	-	9 (52.9%)	-	1 (5.9%)	-	-	17
**total**	**14 (37.8%)**	**3 (8.1%)**	**13 (35.1%)**	**5 (13.5%)**	**2 (5.4%)**	**-**	**-**	**37**

* - = No antibodies of this heavy chain family isolated.
