# Supplementary material for: H3N2 Influenza Infection Elicits More Cross-Reactive and Less Clonally Expanded Anti-Hemagglutinin Antibodies Than Influenza Vaccination
Source: PLoS One. 2011 Oct 19;6(10):e25797. doi: 10.1371/journal.pone.0025797 (PMC3198447; doi:10.1371/journal.pone.0025797)
Supplement: Table S6 — Heavy chain family usage of isolated rmAbs not specific for influenza antigens. (PDF) [file pone.0025797.s019.pdf]

Table S6. Heavy chain family usage of isolated rmAbs not specific for influenza antigens.

Subject	rmAbs Not Specific for Influenza							
	Heavy Chain Family							
	1	2	3	4	5	6	7	Total
	N (%)							N
TIV01	9 (12.5%)	-*	37 (51.4%)	25 (34.7%)	1 (1.4%)	-	-	72
TIV04	-	1 (11.1%)	6 (66.7%)	2 (22.2%)	-	-	-	9
TIV14	5 (33.3%)	-	8 (53.3%)	1 (6.7%)	1 (6.7%)	-	-	15
TIV21	1 (50%)	-	1 (50%)	-	-	-	-	2
TIV24	10 (18.5%)	-	39 (72.2%)	4 (7.4%)	1 (1.9%)	-	-	54
**total**	**25 (16.4%)**	**1 (0.7%)**	**91 (59.9%)**	**32 (21.1%)**	**3 (2%)**	**-**	**-**	**152**
EI02	3 (11.1%)	-	16 (59.3%)	6 (22.2%)	1 (3.7%)	1 (3.7%)	-	27
EI03	15 (15.3%)	6 (6.1%)	51 (52%)	19 (19.4%)	7 (7.1%)	-	-	98
EI05	18 (13%)	5 (3.6%)	77 (55.8%)	28 (20.3%)	8 (5.8%)	2 (1.4%)	-	138
EI07	6 (19.4%)	1 (3.2%)	15 (48.4%)	6 (19.4%)	3 (9.7%)	-	-	31
EI12	5 (9.8%)	-	36 (70.6%)	6 (11.8%)	3 (5.9%)	1 (2%)	-	51
EI13	10 (14.5%)	2 (2.9%)	48 (69.6%)	8 (11.6%)	-	1 (1.4%)	-	69
**total**	**57 (13.8%)**	**14 (3.4%)**	**243 (58.7%)**	**73 (17.6%)**	**22 (5.3%)**	**5 (1.2%)**	**-**	**414**

* - = No antibodies of this heavy chain family isolated.
