# Supplementary material for: H3N2 Influenza Infection Elicits More Cross-Reactive and Less Clonally Expanded Anti-Hemagglutinin Antibodies Than Influenza Vaccination
Source: PLoS One. 2011 Oct 19;6(10):e25797. doi: 10.1371/journal.pone.0025797 (PMC3198447; doi:10.1371/journal.pone.0025797)
Supplement: Table S7 — Kappa chain family usage of isolated influenza-specific rmAbs. (PDF) [file pone.0025797.s020.pdf]

Table S7. Kappa chain family usage of isolated influenza-specific rmAbs.

Subject	Influenza-Specific rmAbs											
	Kappa Chain Family											
	1	2	3	4	5	6	7	1D	2D	3D	6D	Total
	N (%)											N
TIV01	75 (72.1%)	-*	26 (25%)	1 (1%)	-	-	-	2 (1.9%)	-	-	-	104
TIV04	-	-	-	-	-	-	-	-	-	-	-	0
TIV14	-	-	-	-	-	-	-	-	-	-	-	0
TIV21	19 (79.2%)	3 (12.5%)	2 (8.3%)		-	-	-	-	-	-	-	24
TIV24	9 (52.9%)		8 (47.1%)		-	-	-	-	-	-	-	17
**total**	**103 (71%)**	**3 (2.1%)**	**36 (24.8%)**	**1 (0.7%)**	**-**	**-**	**-**	**2 (1.4%)**	**-**	**-**	**-**	**145**
EI02	1 (33.3%)	-	2 (66.7%)	-	-	-	-	-	-	-	-	3
EI03	6 (75%)	-	2 (25%)	-	-	-	-	-	-	-	-	8
EI05	2 (100%)	-	-	-	-	-	-	-	-	-	-	2
EI07	2 (66.7%)	-	1 (33.3%)	-	-	-	-	-	-	-	-	3
EI12	-	-	-	-	-	-	-	-	-	-	-	0
EI13	4 (33.3%)	7 (58.3%)	1 (8.3%)	-	-	-	-	-	-	-	-	12
**total**	**15 (53.6%)**	**7 (25%)**	**6 (21.4%)**	**-**	**-**	**-**	**-**	**-**	**-**	**-**	**-**	**28**

* - = No antibodies of this kappa chain family isolated.
