# Supplementary material for: H3N2 Influenza Infection Elicits More Cross-Reactive and Less Clonally Expanded Anti-Hemagglutinin Antibodies Than Influenza Vaccination
Source: PLoS One. 2011 Oct 19;6(10):e25797. doi: 10.1371/journal.pone.0025797 (PMC3198447; doi:10.1371/journal.pone.0025797)
Supplement: Table S8 — Kappa chain family usage of isolated rmAbs not specific for influenza antigens. (PDF) [file pone.0025797.s021.pdf]

Table S8. Kappa chain family usage of isolated rmAbs not specific for influenza antigens.

Subject	rmAbs Not Specific for Influenza											
	Kappa Chain Family											Total
	1	2	3	4	5	6	7	1D	2D	3D	6D	
	N (%)											N
TIV01	30 (63.8%)	4 (8.5%)	7 (14.9%)	4 (8.5%)	-*	-	-	-	2 (4.3%)	-	-	47
TIV04	4 (80%)	-	1 (20%)	-	-	-	-	-	-	-	-	5
TIV14	3 (27.3%)	1 (9.1%)	4 (36.4%)	2 (18.2%)	-	-	-	-	1 (9.1%)	-	-	11
TIV21	-	-	-	-	-	-	-	-	-	-	-	0
TIV24	28 (66.7%)	3 (7.1%)	6 (14.3%)	3 (7.1%)	-	-	-	1 (2.4%)	-	1 (2.4%)	-	42
**total**	**65 (61.9%)**	**8 (7.6%)**	**18 (17.1%)**	**9 (8.6%)**	**-**	**-**	**-**	**1 (1%)**	**3 (2.9%)**	**1 (1%)**	**-**	**105**
EI02	8 (36.4%)	6 (27.3%)	7 (31.8%)	1 (4.5%)	-	-	-	-	-	-	-	22
EI03	33 (52.4%)	7 (11.1%)	19 (30.2%)	4 (6.3%)	-	-	-	-	-	-	-	63
EI05	35 (38.5%)	9 (9.9%)	38 (41.8%)	7 (7.7%)	-	1 (1.1%)	-	-	1 (1.1%)	-	-	91
EI07	12 (50%)	-	12 (50%)	-	-	-	-	-	-	-	-	24
EI12	24 (57.1%)	7 (16.7%)	8 (19%)	3 (7.1%)	-	-	-	-	-	-	-	42
EI13	28 (57.1%)	11 (22.4%)	8 (16.3%)	2 (4.1%)	-	-	-	-	-	-	-	49
**total**	**140 (48.1%)**	**40 (13.7%)**	**92 (31.6%)**	**17 (5.8%)**	**-**	**1 (0.3%)**	**-**	**-**	**1 (0.3%)**	**-**	**-**	**291**

* - = No antibodies of this kappa chain family isolated.
