# Supplementary material for: H3N2 Influenza Infection Elicits More Cross-Reactive and Less Clonally Expanded Anti-Hemagglutinin Antibodies Than Influenza Vaccination
Source: PLoS One. 2011 Oct 19;6(10):e25797. doi: 10.1371/journal.pone.0025797 (PMC3198447; doi:10.1371/journal.pone.0025797)
Supplement: Table S9 — Lambda chain family usage of isolated influenza-specific rmAbs. (PDF) [file pone.0025797.s022.pdf]

**Table S9.** Lambda chain family usage of isolated influenza-specific rmAbs.

| Subject | Influenza-Specific rmAbs | | | | | | | | | | | |
|---|---|---|---|---|---|---|---|---|---|---|---|---|
| | Lambda Chain Family | | | | | | | | | | | |
| | 1 | 2 | 3 | 4 | 5 | 6 | 7 | 8 | 9 | 10 | 11 | Total |
| | N (%) | | | | | | | | | | | N |
| TIV01 | 33 (47.1%) | 10 (14.3%) | 23 (32.9%) | -* | - | 1 (1.4%) | 2 (2.9%) | - | - | 1 (1.4%) | - | 70 |
| TIV04 | 5 (62.5%) | 1 (12.5%) | 2 (25%) | - | - | - | - | - | - | - | - | 8 |
| TIV14 | 1 (100%) | - | - | - | - | - | - | - | - | - | - | 1 |
| TIV21 | 3 (20%) | 2 (13.3%) | 9 (60%) | - | - | 1 (6.7%) | - | - | - | - | - | 15 |
| TIV24 | 9 (64.3%) | - | 4 (28.6%) | - | - | - | - | - | - | 1 (7.1%) | - | 14 |
| **total** | **51 (47.2%)** | **13 (12.0%)** | **38 (35.2%)** | **-** | **-** | **2 (1.9%)** | **2 (1.9%)** | **-** | **-** | **2 (1.9%)** | **-** | **108** |
| | | | | | | | | | | | | |
| EI02 | 1 (100%) | - | - | - | - | - | - | - | - | - | - | 1 |
| EI03 | 2 (100%) | - | - | - | - | - | - | - | - | - | - | 2 |
| EI05 | 1 (100%) | - | - | - | - | - | - | - | - | - | - | 1 |
| EI07 | - | - | - | - | - | - | - | - | - | - | - | 0 |
| EI12 | - | - | - | - | - | - | - | - | - | - | - | 0 |
| EI13 | 4 (80%) | 1 (20%) | - | - | - | - | - | - | - | - | - | 5 |
| **total** | **8 (88.9%)** | **1 (11.1%)** | **-** | **-** | **-** | **-** | **-** | **-** | **-** | **-** | **-** | **9** |

* - = No antibodies of this lambda chain family isolated.
