# Supplementary material for: H3N2 Influenza Infection Elicits More Cross-Reactive and Less Clonally Expanded Anti-Hemagglutinin Antibodies Than Influenza Vaccination
Source: PLoS One. 2011 Oct 19;6(10):e25797. doi: 10.1371/journal.pone.0025797 (PMC3198447; doi:10.1371/journal.pone.0025797)
Supplement: Table S10 — Lambda chain family usage of isolated rmAbs not specific for influenza antigens. (PDF) [file pone.0025797.s023.pdf]

Table S10. Lambda chain family usage of isolated rmAbs not specific for influenza antigens.

Subject	rmAbs Not Specific for Influenza											
	Lambda Chain Family											
	1	2	3	4	5	6	7	8	9	10	11	Total
	N (%)											N
TIV01	13 (52%)	2 (8%)	7 (28%)	-*	-	-	3 (12%)	-	-	-	-	25
TIV04	-	3 (75%)	1 (25%)	-	-	-	-	-	-	-	-	4
TIV14	-	-	3 (75%)	-	-	-	-	1 (25%)	-	-	-	4
TIV21	-	-	1 (50%)	-	-	-	1 (50%)	-	-	-	-	2
TIV24	-	4 (33.3%)	5 (41.7%)	1 (8.3%)	-	1 (8.3%)	-	1 (8.3%)	-	-	-	12
**total**	**13 (27.7%)**	**9 (19.1%)**	**17 (36.2%)**	**1 (2.1%)**	**-**	**1 (2.1%)**	**4 (8.5%)**	**2 (4.3%)**	**-**	**-**	**-**	**47**
EI02	-	-	1 (20%)	1 (20%)	1 (20%)	1 (20%)	-	1 (20%)	-	-	-	35
EI03	12 (34.3%)	10 (28.6%)	2 (5.7%)	3 (8.6%)	-	1 (2.9%)	4 (11.4%)	3 (8.6%)	-	-	-	
EI05	18 (38.3%)	11 (23.4%)	11 (23.4%)	1 (2.1%)	1 (2.1%)	2 (4.3%)	-	1 (2.1%)	-	2 (4.3%)	-	47
EI07	1 (14.3%)	3 (42.9%)	3 (42.9%)	-	-	-	-	-	-	-	-	7
EI12	1 (11.1%)	1 (11.1%)	1 (11.1%)	1 (11.1%)	5 (55.6%)	-	-	-	-	-	-	9
EI13	8 (40%)	8 (40%)	3 (15%)	-	-	-	-	-	-	1 (5%)	-	20
**total**	**40 (32.5%)**	**33 (26.8%)**	**21 (17.1%)**	**6 (4.9%)**	**7 (5.7%)**	**4 (3.3%)**	**4 (3.3%)**	**5 (4.1%)**	**-**	**3 (2.4%)**	**-**	**123**

* - = No antibodies of this lambda chain family isolated.
