# Supplementary material for: H3N2 Influenza Infection Elicits More Cross-Reactive and Less Clonally Expanded Anti-Hemagglutinin Antibodies Than Influenza Vaccination
Source: PLoS One. 2011 Oct 19;6(10):e25797. doi: 10.1371/journal.pone.0025797 (PMC3198447; doi:10.1371/journal.pone.0025797)
Supplement: Table S11 — Characteristics of cross-reactive rmAbs. (PDF) [file pone.0025797.s024.pdf]

Table S11. Characteristics of cross-reactive rmAbs.

Subject ID	mAb ID	Lineage*	Isotype	V_H	J_H	CDR H3 length	Light chain	V_L	J_L	CDR L3 length	Reactivities[‡]										
											H1 SI	H3 Wisc	TIV07	H1 Bris	H3 Bris	BFla	TIV08	H3 Jobg	H5 Indo	H5 Viet	H1 Cal
TIV01	671	639	G1	4-59	6	19	κ	1-39	1	9	●		○			●		●			
TIV01	1108	N/A	G1	3-74	4	17	κ	3-20	2	10			●		●			●			
TIV01	1122	N/A	G1	3-48	4	14	κ	1-5	1	7	●		○	●							●
TIV01	1213	N/A	G1	1-69	4	11	κ	3-20	2	9	●		○	●			○				●
TIV01	1223	N/A	G1	3-30	4	25	λ	1-40	3	11	●		○	●					●	●	●
TIV01	1290	N/A	G1	3-74	4	12	λ	2-14	2	12	●		○	●							●
TIV01	1314	N/A	G1	3-23	4	16	κ	3-15	2	10	●		○	●							●
TIV01	1823	1945	G1	3-23	3	16	κ	3-15	4	10	●		○	●						●	●
TIV01	1835	1945	G1	3-23	3	16	κ	3-15	4	10	●		○	●						●	●
TIV21	2348	2731	A1	1-2	5	14	κ	1-39	5	9		●			●		○	●			
TIV21	2358	N/A	A1	3-23	4	16	κ	3-15	4	10	●			●			○				●
TIV21	2385	2731	G1	1-2	5	14	κ	1-39	5	9		●			●		○	●			
TIV24	2568	2976	G1	3-13	6	21	κ	3-20	4	10	●			●	●		○				
TIV24	2576	N/A	G1	3-22	3	22	λ	3-1	3	9		●			●		○	●			
EI02	2178	N/A	G1	4-59	5	9	λ	1-40	1	11		●			●			●			
EI03	1941	N/A	G1	1-69	6	16	κ	1-39	4	9		●			●			●			
EI03	1949	N/A	A1	2-70	4	17	λ	1-40	3	11		●			●		○	●			
EI03	1965	N/A	G1	1-18	3	18	κ	1-5	2	11		●	○		●		○	●			●
EI03	2026	N/A	G1	1-69	4	16	κ	3-15	2	11		●	○		●		○	●			
EI07	1894	N/A	G1	1-2	4	14	κ	1-33	4	9		●			●		○	●			
EI13	2207	2569	A1	1-46	3	10	κ	2-24	2	9		●	○	●	●	●	○	●			
EI13	2210	2569	M	1-46	3	10	κ	2-24	2	9		●			●			●			
EI13	2214	N/A	G1	3-23	6	22	κ	3-15	2	10		●			●			●			●
EI13	2217	2569	M	1-46	3	10	κ	2-24	2	9		●	○		●	●		●			
EI13	2221	2569	M	1-46	3	10	κ	2-24	2	9	●	●	○	●	●	●		●			
EI13	2276	N/A	A1	1-2	4	19	λ	1-47	3	11		●			●		○	●			
EI13	2325	N/A	G1	3-11	4	16	κ	2-28	4	17		●	○		●		○	●			

*Lineage ID from Tables S1 and S2. N/A = not applicable

†H1 SI = H1 A/Solomon Islands/03/2006; H3 Wisc = H3 A/Wisconsin/67/2005; TIV07 = trivalent influenza vaccine 2007-2008 season; H1 Bris = H1 A/Brisbane/59/2007; H3Bris = H3 A/Brisbane/10/2007; BFla = HA B/Florida/04/2006; TIV08 = trivalent influenza vaccine 2008-2009 season; H3 Jobg = H3 A/Johannesburg/33/1994; H5 Indo = H5 A/Indonesia/05/2005; H5 Viet = H5 A/Vietnam/1203/2004; H1 Cal = H1 A/California/04/2009.

‡● = reactive with antigen. ○ = reactive with antigen but not counted toward total number of specificities because of overlap with shared antigens.
